# Disrupting the KRAS–SOS1 protein–protein interaction: mechanistic rationale for pan-KRAS pathway suppression and combination therapy

**DOI:** 10.3389/fchem.2026.1808601

**Published:** 2026-04-07

**Authors:** Emadeldin M. Kamel, Sally Mostafa Khadrawy, Mohamed A. M. Ali, Mostafa R. Abukhadra, Nour Y. S. Yassin, Saleh Alkhedhairi, Faris F. Aba Alkhayl, Al Mokhtar Lamsabhi

**Affiliations:** 1 Chemistry Department, Faculty of Science, Beni-Suef University, Beni-Suef, Egypt; 2 Department of Biology, College of Science, Imam Mohammad Ibn Saud Islamic University (IMSIU), Riyadh, Saudi Arabia; 3 Geosciences Department, College of Science, United Arab Emirates University, Al Ain, United Arab Emirates; 4 Physiology Division, Zoology Department, Faculty of Science, Beni-Suef University, Beni-Suef, Egypt; 5 Department of Medical Biosciences, College of Veterinary Medicine, Qassim University, Buraydah, Saudi Arabia; 6 Department of Medical Laboratories, College of Applied Medical Sciences, Qassim University, Buraydah, Saudi Arabia; 7 Departamento de Química and Institute for Advanced Research in Chemical Science (IAdChem), Facultad de Ciencias, Módulo 13, Universidad Autónoma de Madrid, Madrid, Spain

**Keywords:** cancer, KRAS, protein-protein interaction, small molecules, SOS1

## Abstract

Oncogenic KRAS signaling is among the most prevalent drivers in human cancer, yet durable pathway suppression has historically been limited by incomplete target coverage, toxicity constraints for downstream kinase inhibitors, and rapid adaptive rewiring through receptor tyrosine kinase (RTK) feedback. A renewed focus on the KRAS activation cycle has positioned SOS1—an RTK-coupled guanine nucleotide exchange factor—as an attractive upstream node to modulate KRAS output across multiple alleles. By disrupting the KRAS–SOS1 protein–protein interaction (PPI) or otherwise limiting SOS1-mediated nucleotide exchange, SOS1-directed agents reduce RAS-GTP formation and suppress Mitogen-Activated Protein Kinase (MAPK) signaling, while also attenuating feedback-driven rebound that commonly follows MEK/ERK inhibition or allele-specific KRAS targeting. In this review, we summarize the structural and mechanistic basis of RAS–SOS engagement, the emergence of a druggable pocket on SOS1 exploited by modern inhibitors, and the evolution from peptide/interface-mimic approaches to potent small-molecule PPI disruptors. We synthesize key pharmacology across tool compounds and clinical candidates, emphasizing biomarker-linked pharmacodynamic readouts (RAS-GTP and Phosphorylated extracellular signal-regulated kinase (pERK)), context dependence (KRAS allele, RTK tone, and pathway baseline), and on-target validation strategies spanning biophysics, structural biology, and cellular engagement. We then discuss why SOS1 inhibitors act as “multiplier” drugs in rational combinations—particularly with MEK inhibitors and KRAS (G12C) inhibitors—outline expected resistance routes and candidate predictive biomarkers, and review the current clinical landscape for SOS1 inhibitors and combination trial design. Finally, we highlight emerging directions including next-generation, brain-penetrant chemistry and event-driven SOS1 degraders, and propose priorities for translating upstream exchange control into durable patient benefit.

## 1 Introduction

Oncogenic signaling through the RAS family of small GTPases is one of the most frequent molecular drivers of human cancer. A synthesis across major public cancer genomics resources estimates that approximately 19% of patients with cancer harbor activating RAS mutations, with KRAS contributing the majority of this burden and showing marked enrichment in pancreatic, colorectal, and lung adenocarcinoma cohorts (Prior et al., 2012; Prior et al., 2020). Despite this prevalence, RAS proteins were long considered difficult therapeutic targets. The exceptionally high intracellular concentrations of guanine nucleotides, the picomolar affinity of RAS for GDP/GTP, and the historically shallow nature of ligandable surfaces contributed to decades of unsuccessful efforts and the widespread perception that RAS was ‘undruggable’ (Cox et al., 2014).

The clinical activity of covalent KRAS (G12C) inhibitors provided a turning point by demonstrating that direct RAS inhibition can translate into meaningful patient benefit, at least for specific alleles (Skoulidis et al., 2021). However, KRAS (G12C) represents only a subset of the broader KRAS-mutant disease burden, and adaptive pathway reactivation can limit depth and durability of response. These limitations have strengthened interest in complementary strategies that modulate KRAS signaling by targeting key regulatory steps that are shared across KRAS alleles (Cox et al., 2014; Prior et al., 2020). A central control point is KRAS nucleotide loading. KRAS cycles between an inactive GDP-bound state and an active GTP-bound state, with guanine nucleotide exchange factors (GEFs) accelerating GDP release to enable GTP binding. SOS1 (Son of Sevenless 1) is a principal RAS GEF in many epithelial contexts and forms a productive complex with KRAS that couples upstream receptor tyrosine kinase signaling to RAS activation (Boriack-Sjodin et al., 1998). Seminal structural studies defined how the SOS catalytic module engages RAS to promote nucleotide exchange and revealed additional layers of regulation that are directly relevant to drug design. In particular, SOS can be allosterically stimulated by RAS bound to Guanosine Triphosphate (active form of RAS) (RAS-GTP) binding at a distal site, creating a positive feedback mechanism, and the full-length protein adopts autoinhibited conformations that can be relieved by membrane recruitment and signaling inputs (Boriack-Sjodin et al., 1998; Margarit et al., 2003; Sondermann et al., 2004).

These mechanistic insights motivated the idea that interrupting the KRAS-SOS1 protein-protein interaction (PPI) could suppress KRAS. GTP formation upstream of effector engagement, offering a potential route to broader (allele-agnostic) control of KRAS pathway output. Early work established feasibility using engineered, hydrocarbon-stapled peptides derived from SOS1 helices that could disrupt SOS1-KRAS engagement and inhibit KRAS-driven signaling in cells (Leshchiner et al., 2015). The field then advanced rapidly with the discovery of drug-like small molecules that bind a pocket within the SOS1 catalytic domain and sterically prevent productive KRAS binding, functionally disrupting the KRAS-SOS1 PPI. BAY-293 became a widely used chemical probe that enabled systematic interrogation of SOS1 dependence across RAS-pathway contexts (Hillig et al., 2019). Subsequent optimization yielded orally bioavailable inhibitors with robust pathway pharmacology. BI-3406 was reported as a potent and selective SOS1 inhibitor that reduces formation of GTP-loaded RAS and, importantly, attenuates feedback reactivation induced by mitogen-activated protein kinase (also called MAPK/ERK Kinase) (MEK) inhibition, supporting vertical combination strategies in KRAS-driven tumors (Hofmann et al., 2021).

More recently, MRTX0902 was developed as a potent, selective, brain-penetrant SOS1 binder that disrupts the SOS1:KRAS PPI and enhances antitumor activity when combined with the KRAS (G12C) inhibitor adagrasib (MRTX849) in preclinical models (Ketcham et al., 2022). Translational efforts have progressed into early-phase clinical evaluation. BI 1701963 (a SOS1 KRAS interaction inhibitor) is being studied as monotherapy and in combination with the MEK inhibitor trametinib, and MRTX0902 is being evaluated alone and in combination with adagrasib in advanced solid tumors with KRAS-MAPK pathway alterations (Sütcüoğlu et al., 2025; Tria et al., 2023).

In this review, we summarize the biological rationale for targeting the KRAS-SOS1 interface, highlight the evolution of chemical modalities that disrupt this PPI, and discuss pharmacology, combination strategies, resistance mechanisms, and key considerations for clinical translation.

## 2 RAS mutations in the cancer landscape

**FIGURE 1:**
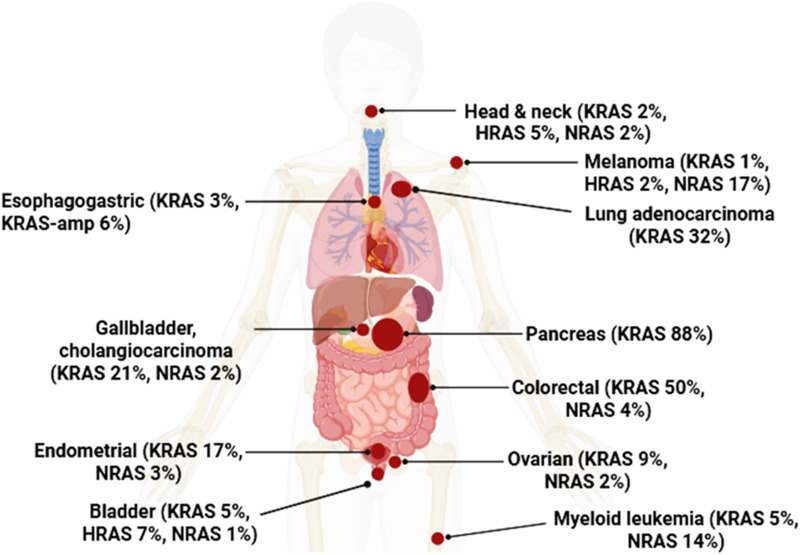
Frequency of RAS alterations across human cancers. Schematic overview of the prevalence of KRAS, NRAS, and HRAS mutations in selected tumor types. Percentages indicate the proportion of cases harboring mutations in each RAS isoform for the indicated cancer, with KRAS amplification (KRAS-amp) shown where applicable.

RAS genes and their isoforms were recognized as oncogenes with tumor-forming potential more than 30 years ago (Cooper, 1982; Parada et al., 1982; Santos et al., 1982; Taparowsky et al., 1982). Mutational activation of RAS occurs in roughly one-third of human cancers, making it among the most frequent oncogenic alterations. The most commonly mutated isoform is KRAS, followed by NRAS and HRAS (Baines et al., 2011; Pylayeva-Gupta et al., 2011). Most RAS alterations are single–base substitutions at codons 12, 13, and 61 (G12, G13, and Q61), which lock RAS proteins in a constitutively active state (Hobbs et al., 2016). In KRAS, mutations occur mainly at G12 (about 83% of all KRAS mutations), whereas NRAS and HRAS show more variable distributions across all three sites (Simanshu et al., 2017). Substitutions at codon 12 or 13 with almost any amino acid other than proline introduce steric hindrance that prevents hydrolysis of RAS-GTP to RAS-GDP (Simanshu et al., 2017). Consequently, aberrant upregulation of RAS signaling and downstream effectors drives many hallmark cancer traits, including increased proliferation, resistance to apoptosis, metabolic reprogramming, remodeling of the tumor microenvironment, immune evasion, and metastasis (Hobbs et al., 2016; Karnoub and Weinberg, 2008; Knickelbein and Zhang, 2015; Pylayeva-Gupta et al., 2011; Rodenhuis, 1992). Figure 1 summarizes mutation frequencies and tumor associations across the RAS isoforms.

## 3. KRAS remains a central oncogenic driver—why upstream control still matters

### 3.1 KRAS cycling and pathway output: MAPK/ERK, PI3K, and beyond

**FIGURE 2:**
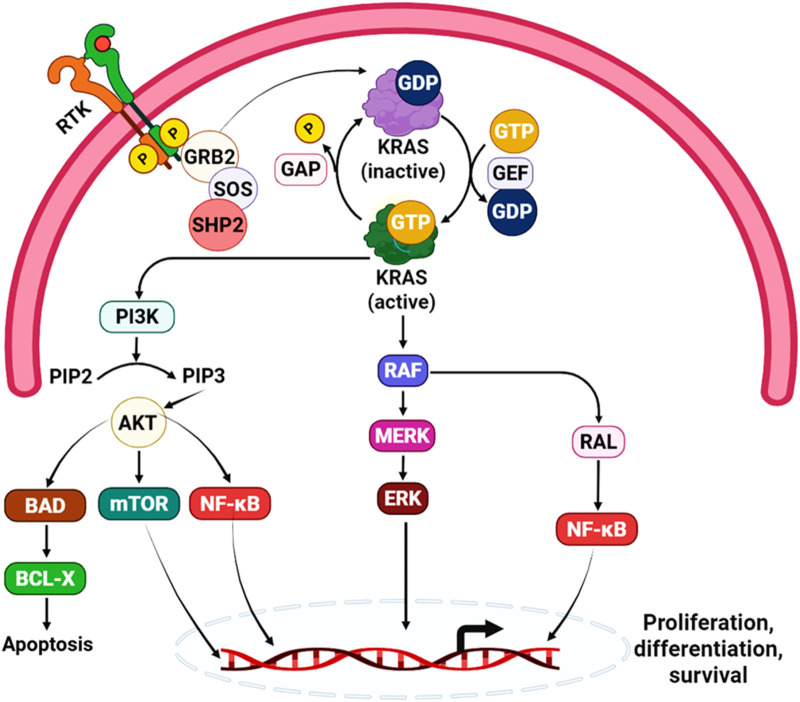
Overview of KRAS activation and downstream RAS signaling. Ligand-induced activation of receptor tyrosine kinases (RTKs) recruits the adaptor growth factor receptor-bound Protein 2 (GRB2) and the guanine nucleotide exchange factor SOS (often facilitated by SHP2) to the plasma membrane, where SOS promotes GDP–GTP exchange on membrane-bound KRAS to generate active, GTP-loaded KRAS. KRAS activity is normally controlled by cycling between inactive (GDP-bound) and active (GTP-bound) states, regulated by GEFs (which stimulate nucleotide exchange) and GAPs (which accelerate GTP hydrolysis). Oncogenic KRAS mutations impair this regulation, leading to sustained accumulation of active KRAS and persistent activation of downstream effector pathways, including RAF–MEK–ERK (MAPK) and PI3K–AKT–mTOR, thereby promoting tumor cell proliferation, differentiation, and survival.

KRAS sits at the center of multiple oncogenic signaling networks and remains one of the most consequential driver genes in human cancer (Ostrem and Shokat, 2016; Prior et al., 2020). Even in tumors driven by mutant KRAS, pathway output is not simply a fixed “always-on” state: it reflects the fraction of KRAS in its active, GTP-bound conformation over time, plus the availability of effector proteins and feedback circuits that reshape signal flow (Kolch et al., 2023; Mozzarelli et al., 2024). This dynamic view is central to understanding why disrupting the KRAS–SOS1 interface can remain therapeutically relevant even in the era of direct KRAS inhibitors (Hofmann et al., 2021; Isermann et al., 2025) (Figure 2).

At the biochemical level, RAS proteins function as nucleotide-dependent molecular switches. Guanine nucleotide exchange factors (GEFs), including SOS1, catalyze GDP release and promote GTP loading, whereas GTPase-activating proteins (GAPs) accelerate GTP hydrolysis to terminate signaling (Kolch et al., 2023; Ostrem and Shokat, 2016). Oncogenic KRAS mutations often weaken intrinsic and/or GAP-stimulated hydrolysis, shifting the steady-state toward KRAS-GTP; however, this effect is allele dependent, and many mutant proteins still continue to cycle between nucleotide states rather than remaining constitutively GTP bound. As a result, the fraction of KRAS that remains GDP loaded is shaped by the balance between intrinsic exchange, intrinsic hydrolysis, GAP-mediated hydrolysis, and upstream receptor tyrosine kinase (RTK) input, all of which influence the extent to which a given mutant remains susceptible to SOS1-dependent nucleotide exchange (Fedele et al., 2021; Kolch et al., 2023; Ostrem and Shokat, 2016) (Figure 2). This is especially evident for KRAS (G12C), and in preclinical systems also KRAS (G12D), where retention of a therapeutically meaningful GDP-bound fraction helps explain the activity of inhibitor classes that depend on access to the inactive state, while also providing a rationale for combining such agents with SOS1 inhibition to further bias nucleotide occupancy away from KRAS-GTP (Fedele et al., 2021; Hallin et al., 2022; Jänne et al., 2022). More broadly, these biochemical considerations suggest that the clinical positioning of SOS1 inhibitors will likely differ across KRAS alleles: mutants with greater residual nucleotide cycling may remain more sensitive to upstream exchange blockade, whereas alleles with more severely impaired hydrolysis may be less dependent on SOS1. This may also be relevant for non-canonical settings such as KRAS (G12R), where distinct nucleotide-cycling behavior could influence the degree of SOS1 dependence and, therefore, the expected benefit of SOS1-directed strategies (Fedele et al., 2021; Hallin et al., 2022; Jänne et al., 2022).

Once GTP-bound, KRAS engages several effector families that together account for most oncogenic pathway output. The RAF–MEK–ERK (MAPK) cascade is a dominant branch controlling proliferation and differentiation programs, while class I PI3Ks link KRAS to AKT–mTOR signaling, metabolic rewiring, and survival (Mozzarelli et al., 2024; Ostrem and Shokat, 2016). In parallel, KRAS can activate Ral guanine nucleotide exchange factors (RalGEFs) to drive RAL signaling and additional phenotypes linked to growth, vesicle trafficking, and invasion (Mozzarelli et al., 2024; Ostrem and Shokat, 2016) (Figure 2). Importantly, the amplitude and duration of ERK signaling—and the integration of MAPK with phosphoinositide 3-kinase (PI3K) and other branches—are shaped by layered negative feedback (e.g., ERK-dependent suppression of upstream RTK signaling and adaptor function), which can be rapidly relieved by targeted therapies.^15^

### 3.2 Where SOS1 fits relative to KRAS allele-specific inhibitors and downstream kinase inhibitors

**FIGURE 3:**
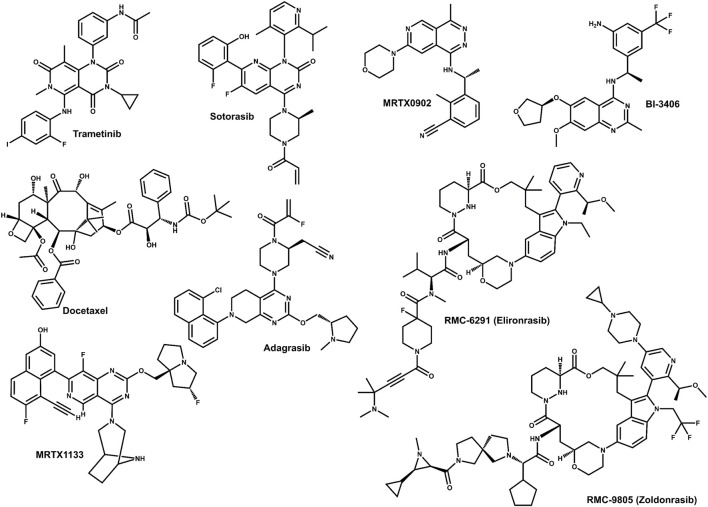
Chemical structures of compounds used to illustrate key therapeutic modalities along the RAS pathway, including downstream pathway targeting (trametinib, MEK inhibitor; docetaxel, chemotherapy comparator), allele-selective KRAS inhibition (sotorasib and adagrasib, covalent KRAS^G12C^(OFF) inhibitors; MRTX1133, non-covalent KRAS^G12D^ inhibitor), proximal pathway control via SOS1 (MRTX0902 and BI-3406, KRAS–SOS1 pathway/SOS1 inhibitors), and active-state “RAS(ON)” inhibition (RMC-6291, KRAS^G12C(ON)^; RMC-9805, KRAS^G12D(ON)^).

Historically, the most clinically mature approach to KRAS-driven cancers was to target downstream kinases in the MAPK pathway. However, MEK (and later ERK) inhibitor monotherapy has typically delivered modest and/or transient benefit in KRAS-mutant solid tumors, in part because toxicity constrains sustained pathway shutdown and because tumors rapidly rewire signaling via feedback (Ahmed et al., 2019; Blumenschein et al., 2015). For example, in a randomized phase II study in advanced KRAS-mutant non-small-cell lung cancer (NSCLC), trametinib did not improve outcomes compared with docetaxel (Figure 3) (Blumenschein et al., 2015). Mechanistically, MEK inhibition can relieve ERK-dependent negative feedback, inducing RTK signaling and restoring RAS–MAPK activity; in KRAS-mutant lung cancer models, epithelial-to-mesenchymal state has been shown to influence which RTKs dominate this rebound response (Kitai et al., 2016). Similar adaptive programs can also limit ERK inhibitor efficacy via proximal signaling nodes such as Src homology-2 domain-containing protein tyrosine phosphatase-2 (encoded by *PTPN11*) (SHP2), underscoring the value of combining distal kinase inhibitors with upstream pathway control (Ahmed et al., 2019).

**FIGURE 4:**
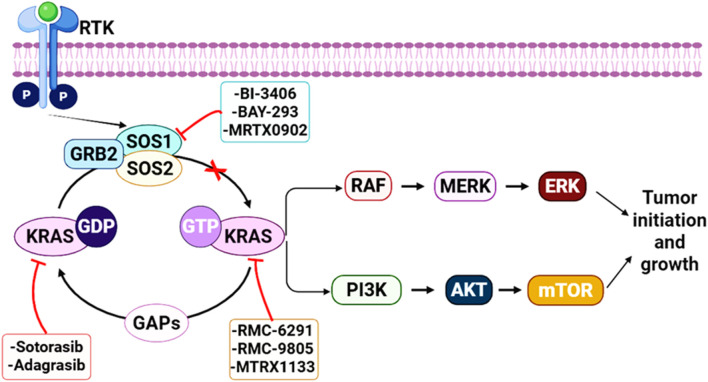
Therapeutic intervention points in KRAS-driven signaling. Activated receptor tyrosine kinases (RTKs) recruit the adaptor GRB2 and the guanine-nucleotide exchange factors SOS1/SOS2, promoting GDP–GTP exchange and generating active, GTP-loaded KRAS; GAPs stimulate GTP hydrolysis to return KRAS to the inactive GDP-bound state. Oncogenic KRAS sustains downstream signaling through the RAF–MEK–ERK and PI3K–AKT–mTOR pathways, supporting tumor initiation and growth. Representative strategies include direct KRAS inhibitors that engage specific nucleotide states (e.g., sotorasib and adagrasib for KRAS^G12C^ (GDP/“OFF”) and agents targeting active-state or non-G12C KRAS such as RMC-6291, RMC-9805, and MRTX1133), as well as upstream blockade of KRAS activation via SOS1 inhibition (e.g., BI-3406, BAY-293, MRTX0902) to limit nucleotide exchange and dampen pathway reactivation.

Direct, allele-selective KRAS inhibitors have transformed the landscape but are not (yet) universally applicable. Sotorasib and adagrasib (Figure 3) are covalent KRASG12C(OFF) inhibitors that bind the inactive, GDP-bound conformation and have received U.S. FDA accelerated approvals for previously treated KRASG12C-mutant NSCLC (Jänne et al., 2022; Mausey and Halford, 2024; Parums, 2022; Skoulidis et al., 2021). Because these agents preferentially engage KRASG12C-GDP, the rate of nucleotide cycling and the strength of upstream RTK→GEF signaling directly influence both drug engagement and the pace of pathway reactivation (Fedele et al., 2021; Ryan et al., 2022). In cell and animal models, proximal pathway inhibition (e.g., SHP2 blockade) can increase KRASG12C GDP occupancy and suppress RTK-feedback signaling, thereby enhancing KRASG12C inhibitor activity and delaying adaptive resistance (Fedele et al., 2021; Ryan et al., 2022) (Figure 4).

SOS1 inhibition targets this same proximal “re-loading” axis but at the level of guanine nucleotide exchange on RAS. The rationale is twofold. First, by limiting GDP→GTP exchange, SOS1 inhibitors can reduce the pool of active RAS across multiple KRAS alleles and can blunt the rapid rebound in RAS–MAPK signaling that follows pharmacologic suppression downstream (Hofmann et al., 2021; Kitai et al., 2016; Kolch et al., 2023). Second, in settings where direct KRAS inhibitors favor a particular nucleotide state, reducing exchange can bias KRAS toward that state and improve the depth and durability of pathway inhibition (Fedele et al., 2021). Consistent with this logic, SOS1 inhibition has been reported to enhance the efficacy of KRASG12C inhibitors and delay resistance in lung adenocarcinoma models. ^25^ In addition, the SOS1 inhibitor MRTX0902 (Figure 3) (a KRAS–SOS1 PPI disruptor) has shown preclinical anti-tumor activity and synergistic interactions with KRAS and MEK inhibitors in KRAS-driven models, supporting SOS1 as a rational combination partner rather than a stand-alone substitute for direct or distal pathway blockade (Sudhakar et al., 2024).

The positioning of SOS1 inhibition becomes even more relevant as allele-selective KRAS pharmacology expands beyond G12C. Preclinical agents such as the non-covalent KRASG12D inhibitor MRTX1133 (Figure 3) show that other common KRAS alleles can be directly targeted, but early studies also highlight bypass and feedback dependencies that may require combination strategies (Hallin et al., 2022; Isermann et al., 2025). Notably, BI-3406 (Figure 3) synergized with MRTX1133 in an immunocompetent KRASG12D-driven lung adenocarcinoma model, providing direct *in vivo* evidence that SOS1 inhibition can complement emerging non-G12C KRAS inhibitors (Baltanás et al., 2025). In parallel, “RAS(ON)” inhibitors that target active, GTP-bound oncogenic RAS are being evaluated in early-phase trials (e.g., RMC-6291 for KRASG12C(ON) and RMC-9805 (Figure 3) for KRASG12D (ON)), expanding the spectrum of nucleotide-state-directed therapies (Li et al., 2026; Solanki et al., 2026). Across these modalities, SOS1 sits upstream of KRAS at a convergence point for RTK-driven pathway restoration, making KRAS–SOS1 PPI disruption a compelling strategy to deepen pathway suppression and counter adaptive rewiring when paired with direct KRAS inhibitors or downstream kinase inhibitors (Isermann et al., 2025; Kolch et al., 2023; You et al., 2016) (Figure 4). This concept may be especially relevant in KRAS (G12D)-mutant hematologic malignancies, where oncogenic KRAS has been reported to drive hyperactivation of wild-type NRAS and HRAS, and genetic loss of SOS1 attenuated this effect, reduced downstream ERK signaling, and improved disease phenotypes, suggesting that the benefit of SOS1 inhibition may extend beyond direct suppression of mutant KRAS alone (You et al., 2018).

## 4 SOS1 biology in the KRAS activation cycle

SOS1 is the canonical receptor-proximal Ras guanine nucleotide exchange factor (RasGEF) that links activated receptor tyrosine kinases (RTKs) to Ras activation by coupling phosphotyrosine signaling to nucleotide exchange on membrane-bound Ras (Chardin et al., 1993). This function is central to how growth-factor cues regulate the Ras–MAPK pathway in normal cells, but it is also highly relevant in KRAS-mutant cancers: even when KRAS is oncogenically mutated, tumors remain embedded in RTK-rich microenvironments, retain wild-type Ras paralogs, and can exploit upstream signaling to amplify pathway output, diversify effector engagement, and adapt to targeted therapy. Understanding how SOS1 is regulated at the membrane and how its activity differs from SOS2 is therefore essential for rational design and clinical positioning of KRAS–SOS1 PPI disruptors.

### 4.1 SOS1 as a guanine nucleotide exchange factor (GEF): catalytic logic

At the protein level, SOS1 is a multi-domain enzyme whose regulatory N-terminus is fused to a conserved catalytic core. The catalytic unit is formed by the RAS exchanger motif (REM) and the CDC25 homology domain, whereas the N-terminus contains a histone-fold module followed by Dbl homology (DH) and pleckstrin homology (PH) domains, and the C-terminus contains a proline-rich region that binds the SH3 domains of Grb2 (Christensen et al., 2016; Qian et al., 1998; Sondermann et al., 2004). This architecture allows SOS1 to integrate receptor-driven recruitment signals with membrane-dependent activation cues and allosteric feedback.

Structural analyses of RAS–SOS complexes established the core catalytic logic: Ras substrate (classically studied with HRAS but conserved across RAS isoforms) binds the CDC25 domain, where a conserved “helical hairpin” from SOS intrudes into the Ras switch region, destabilizing the nucleotide- and Mg2+-binding environment and dramatically accelerating GDP release (Boriack-Sjodin et al., 1998). Once GDP dissociates, abundant cellular GTP rapidly rebinds to produce RAS-GTP; SOS1 does not “load” GTP, it catalyzes GDP exit by lowering the activation barrier for nucleotide dissociation (Boriack-Sjodin et al., 1998). The REM domain does not supply the exchange chemistry directly; rather, it organizes the catalytic core and—critically—creates an allosteric Ras-binding site at the REM–CDC25 interface (Freedman et al., 2006; Margarit et al., 2003).

A defining feature of SOS-family RAS GEFs is the presence of this second Ras-binding site (the allosteric site). Binding of RAS-GTP at the allosteric site increases nucleotide exchange at the catalytic site, providing structural support for a positive feedback model in which initial Ras activation can trigger a self-amplifying burst of exchange activity (Freedman et al., 2006; Margarit et al., 2003). Conformational analyses further suggest that allosteric Ras binding stabilizes an “open,” exchange-competent arrangement of the REM–CDC25 core (Rojas and Santos, 2006). Importantly, because KRAS-mutant tumors still contain wild-type KRAS/NRAS/HRAS and can generate RAS-GTP through RTK inputs, the allosteric activation loop provides a mechanistic rationale for why SOS1 remains a relevant upstream control point even in the presence of mutant KRAS.

### 4.2 Autoinhibition and feedback regulation (including RAS-GTP-mediated effects)

Full-length SOS1 is strongly autoinhibited in the cytosol. In the autoinhibited state, N-terminal regulatory elements restrict productive membrane engagement and sterically hinder the allosteric Ras-binding pocket, preventing SOS1 from efficiently entering the positive-feedback regime (Gureasko et al., 2010; Sondermann et al., 2004; Yadav and Bar-Sagi, 2010). Functional dissection showed that the DH and PH domains (and adjacent N-terminal elements) are not merely passive appendages: they enforce autoinhibition and couple membrane recruitment to enzymatic activation, so that SOS1 activity is conditional on receiving the correct upstream and membrane-context cues (Christensen et al., 2016; Qian et al., 1998).

Relief of autoinhibition occurs through coordinated inputs. First, activated RTKs (or phosphotyrosine adaptors) recruit Grb2 via its SH2 domain; Grb2 then binds the SOS1 proline-rich region through its SH3 domains, translocating SOS1 to the plasma membrane (Chardin et al., 1993). Second, membrane proximity enables regulatory-domain interactions with acidic phospholipids and Ras itself. Structural snapshots explain why this is necessary: the DH/PH module can occlude the allosteric pocket in the catalytic core, such that allosteric Ras binding becomes a key “licensing” step for full activation (Sondermann et al., 2004). Consistent with this, studies of SOS1 regulation by the histone-fold domain indicate that the N-terminus gates access to the allosteric site and helps set the threshold for activation (Gureasko et al., 2010; Yadav and Bar-Sagi, 2010).

Once a small pool of RAS-GTP is generated, RAS-GTP binding at the allosteric site can amplify SOS1 activity (feedback activation), increasing the rate at which additional Ras molecules are converted to RAS-GTP (Freedman et al., 2006; Margarit et al., 2003). Membrane-based reconstitution and single-molecule experiments add a critical kinetic dimension: membrane-associated SOS can behave processively—remaining on the membrane for extended periods and activating many Ras molecules—such that receptors can “seed” a long-lived active state that persists beyond the initial recruitment event (Christensen et al., 2016). In cells, termination of this membrane-trapped, active state can require active removal mechanisms (for example, endocytic trafficking), supporting a model in which SOS activation is not simply a rapidly reversible binding equilibrium but can include a quasi–one-way membrane engagement step (Christensen et al., 2016).

Downstream feedback loops provide additional layers of control. A well-characterized negative feedback is ERK/MAPK-mediated phosphorylation of SOS1 at multiple sites, which reduces SOS1 association with Grb2 and attenuates further Ras activation, helping shape the amplitude and duration of MAPK pathway signaling (Corbalan-Garcia et al., 1996). Together, positive feedback (RAS-GTP allosteric activation) and negative feedback (ERK-mediated desensitization) allow SOS1 to function as an input-sensitive, self-amplifying, yet self-limiting node in the KRAS activation cycle—properties that are directly relevant when considering pharmacologic disruption of the KRAS–SOS1 interface.

### 4.3 SOS1 vs. SOS2: redundancy, tissue context, and therapeutic implications

Mammals express two closely related SOS proteins (SOS1 and SOS2) with similar domain architecture and the capacity to activate RAS, yet genetic studies show important nonredundancy. SOS1 is essential for normal development—its loss causes embryonic lethality driven in part by placental defects—whereas SOS2 loss is compatible with normal mouse growth and fertility (Esteban et al., 2000; Qian et al., 1998). In growth-factor signaling, both proteins can couple RTKs to RAS activation, but they differ in signaling kinetics and complex assembly: in EGF-stimulated systems, SOS1 supports both acute and sustained ERK signaling, while SOS2-dependent signals are predominantly short-term, correlating with reduced long-term association of SOS2 with upstream signaling complexes (Qian et al., 1998). At the biochemical level, SOS1 and SOS2 also display measurable differences in their interactions with Grb2; for example, Sos2 can bind Grb2 with higher apparent affinity than Sos1 *in vitro* and in cells (Yang et al., 1995). These observations have implications not only for pathway biology but also for clinical safety. On the one hand, the developmental requirement for SOS1 argues that this paralog has important nonredundant physiologic functions, raising the possibility that deep or prolonged on-target inhibition could narrow the therapeutic window. On the other hand, the relative dispensability of SOS2 suggests that selective SOS1 inhibition may still offer a more favorable therapeutic index than broader SOS-family suppression, because some physiologic Ras-exchange capacity may be preserved in normal tissues. Importantly, however, embryonic knockout phenotypes do not map directly onto adult pharmacologic inhibition, so the tolerability consequences of selective SOS1 blockade will need to be defined empirically, particularly for chronic dosing and combination regimens.

In oncogenic RAS contexts, the relative contribution of SOS1 and SOS2 is strongly model- and tissue-dependent. In a KRASG12D-driven lung adenocarcinoma mouse model, genetic SOS1 ablation produced marked and durable benefits—including reduced tumor burden and extended survival—whereas SOS2 ablation offered more limited protection, particularly at later stages; SOS1 loss also altered features of the tumor microenvironment (Baltanás et al., 2025). Conversely, in transformation models and in some human KRAS-mutant cell settings, SOS2 can be a critical mediator of mutant KRAS-driven phenotypes by promoting EGFR-dependent activation of wild-type RAS and PI3K–AKT signaling; SOS2 loss reduced Protein Kinase B (PKB) (AKT) phosphorylation and synergized with MEK inhibition to suppress transformed growth (Sheffels et al., 2018). Reviews of SOS biology emphasize that this division of labor can reflect tissue-specific expression, receptor wiring, and pathway dependencies, implying that neither isoform’s contribution can be assumed *a priori* across KRAS-driven disease contexts (Baltanás et al., 2025).

These observations have direct therapeutic implications for KRAS–SOS1 PPI disruption. First, the developmental dispensability of SOS2 and the frequent dominance of SOS1 in sustaining KRAS-driven tumor progression *in vivo* support the tractability of selectively targeting SOS1 in cancer (Baltanás et al., 2025; Esteban et al., 2000). Second, partial redundancy implies that SOS2 expression or pathway rewiring could provide an escape route in some settings—particularly where PI3K–AKT signaling is a key dependency—arguing for biomarker-aware strategies and rational combinations (Baltanás et al., 2025; Sheffels et al., 2018). Practically, this strengthens the rationale for evaluating SOS1 inhibitors (including KRAS–SOS1 PPI disruptors) both as monotherapy candidates in SOS1-dominant contexts and as components of combination regimens designed to blunt compensatory inputs (for example, with RTK/SHP2 blockade or downstream pathway inhibitors) when isoform redundancy or pathway bifurcation becomes limiting.

## 5 The KRAS–SOS1 interface as a drug target: structural and mechanistic basis

A central challenge in “drugging” KRAS is that its oncogenic activity is sustained not only by mutation but also by continual pathway rewiring and adaptive feedback. Because SOS1 is the principal RAS GEF connecting receptor-proximal signals to RAS activation, the KRAS–SOS1 interaction provides a mechanistically grounded intervention point: preventing formation of a productive exchange complex limits GDP release and can suppress both basal and feedback-driven reloading of RAS with GTP (Boriack-Sjodin et al., 1998; Hillig et al., 2019; Hofmann et al., 2021; Margarit et al., 2003). The structural biology of RAS–SOS engagement also explains why this interface is more tractable than many PPIs—SOS1 presents ligandable cavities adjacent to the RAS-binding surface that can be exploited to block complex formation (Burns et al., 2014; Hillig et al., 2019; Hofmann et al., 2021).

### 5.1 Structural snapshots of RAS–SOS engagement (switch regions and nucleotide exchange mechanics)

X-ray structures of RAS bound to the catalytic region of SOS1 captured the exchange intermediate in which RAS is stabilized in a nucleotide-free state (for example, the H-RAS–SOS1 complex solved by Boriack-Sjodin and colleagues) (Boriack-Sjodin et al., 1998). In these structures, RAS docks onto the SOS1 CDC25 domain primarily through the switch I and switch II regions, and SOS1 promotes nucleotide release by inserting a helical hairpin (the “catalytic helix”) into the nucleotide-binding site. This interaction displaces switch I and perturbs coordination of the bound nucleotide and Mg2+, sharply weakening GDP affinity and favoring the transient, nucleotide-free state required for reloading with GTP (Boriack-Sjodin et al., 1998). Subsequent structures showed how RAS-GTP can bind a spatially distinct allosteric site on SOS1 to enhance exchange activity, providing a structural basis for positive feedback: RAS-GTP engagement stabilizes an active SOS1 conformation and increases the rate of SOS-catalyzed nucleotide exchange on a second RAS molecule (Margarit et al., 2003).

**FIGURE 5:**
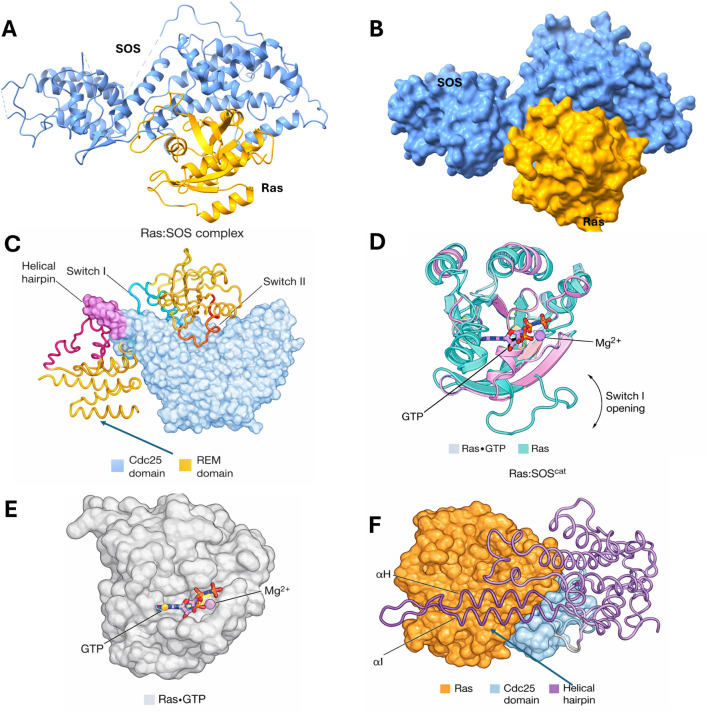
Summarizes the structural mechanism by which SOS engages RAS and catalyzes nucleotide exchange. In the nucleotide-free RAS-SOS^cat^ complex (PDB 1BKD), RAS binds the SOS Cdc25 catalytic domain through an extensive interface dominated by Switch I and Switch II **(A–C)** (Bandaru et al., 2019). The defining catalytic feature is the protruding SOS helical hairpin, which inserts between Switch I and Switch II and pries Switch I away from the RAS core—effectively opening the nucleotide-binding pocket and disrupting productive coordination of the nucleotide phosphates and Mg^2+^ **(D,F)**. By comparison, GTP-bound RAS adopts a more closed Switch I configuration (**(E)**; PDB 5P21), highlighting how SOS-induced “Switch I peeling” stabilizes a transient, nucleotide-free exchange intermediate that is poised for re-loading (Bandaru et al., 2019).

Figure 5 Structural basis of RAS engagement by SOS and the mechanism of nucleotide exchange (Bandaru et al., 2019). (A) Ribbon representation of the RAS-SOS catalytic complex (PDB 1BKD), with SOS shown in blue and RAS in gold. (B) Surface representation of the same RAS-SOS complex (PDB 1BKD). (C) Structural overview of the RAS-SOS^cat^ complex (PDB 1BKD) illustrating how SOS stabilizes the nucleotide-free form of RAS through an extensive interface that engages Switch I and Switch II; the REM and Cdc25 domains of SOS are indicated. (D) Conformational change associated with exchange: insertion of the SOS helical hairpin into RAS promotes nucleotide release primarily by peeling Switch I away from the RAS core, opening the nucleotide-binding site and disrupting Mg^2+^/nucleotide coordination. (E) Structure of RAS-GTP (PDB 5P21) highlighting the bound nucleotide and Mg^2+^. (F) Model of the exchange-active configuration showing the SOS Cdc25 helical hairpin inserted between Switch I and Switch II, widening the active site and facilitating expulsion of the bound nucleotide. Abbreviations: REM, RAS exchanger motif; GTP, guanosine triphosphate (Bandaru et al., 2019).

Together, these snapshots establish two key mechanistic points that are directly relevant to inhibitor design. First, SOS1-driven activation requires a stereochemically precise docking geometry that couples switch-region engagement to positioning of the catalytic helix; partial engagement is not productive. Second, because the exchange complex is transient and involves extensive remodeling of flexible switch loops, the effective “interface” includes dynamic pockets and grooves that can be targeted indirectly—by ligands that occupy SOS1 cavities adjacent to the RAS-contact surface and thereby prevent the productive, nucleotide-exchange-competent complex from forming (Boriack-Sjodin et al., 1998; Margarit et al., 2003).

### 5.2 The SOS1 “druggable pocket” exploited by modern inhibitors: what it is and why it works

Multiple studies converged on a ligandable pocket on the SOS1 CDC25 domain located immediately adjacent to the catalytic RAS-binding surface. Early chemical biology work demonstrated that small molecules can occupy a hydrophobic pocket in the SOS CDC25 domain near the RAS switch II region within a RAS-SOS complex, validating this region as “ligandable” in a physiologically relevant assembly (Burns et al., 2014). Notably, these first ligands increased the rate of SOS-catalyzed nucleotide exchange *in vitro* (Burns et al., 2014), emphasizing that binding in this area can tune (rather than simply abolish) SOS1’s catalytic function—an idea later leveraged to obtain true PPI inhibitors.

**FIGURE 6:**
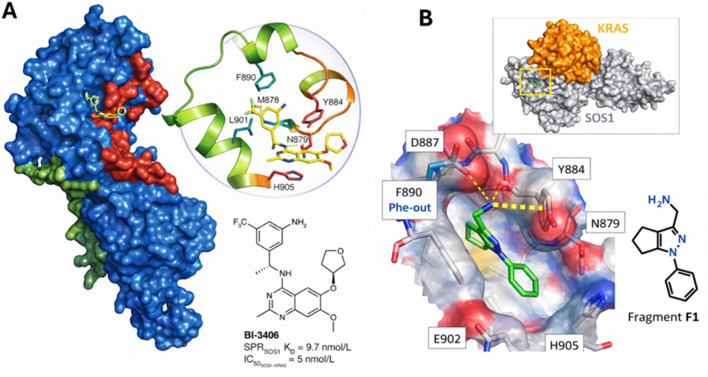
Structural definition of the SOS1 CDC25 “druggable pocket” and how ligands perturb RAS–SOS engagement. **(A)** Co-crystal structure of the SOS1 CDC25 domain with the SOS1 inhibitor BI-3406 bound in a pocket adjacent to the catalytic RAS-binding surface (ligand highlighted in yellow; SOS1 shown as a surface). The catalytic RAS interaction region (mapped from PDB 1NVU) and the distal allosteric RAS site are indicated for spatial context. The zoomed view summarizes key BI-3406 contacts within the pocket and illustrates that residues contributing to catalytic RAS binding overlap with, or lie immediately beside, the inhibitor footprint—providing a structural explanation for steric/competitive interference with RAS docking. The chemical structure of BI-3406 and representative potency values are shown. *Adapted from* Hofmann et al. (2021). **(B)** Structure of a fragment ligand (F1) bound within the KRAS^G12C^–SOS1^cat^ assembly. The upper inset shows the overall complex and the position of the fragment-binding site (boxed), while the enlarged view depicts the local interaction network, including hydrogen-bonding contacts (thin dashed lines) and a cation–π interaction (thick dashed line). *Adapted from* Hillig et al. (2019).

Modern KRAS–SOS1 disruptors bind this same general neighborhood but are optimized to block RAS docking. For example, the tool compound BAY-293 was discovered as a potent SOS1 inhibitor that blocks RAS activation by disrupting the RAS–SOS1 interaction; structural and biochemical analyses place the inhibitor in a surface pocket on SOS1 immediately adjacent to the KRAS binding site (Hillig et al., 2019). The clinical-stage chemotype BI-3406 provides an especially clear structure–mechanism link: co-crystal structures show BI-3406 bound in a pocket next to the SOS1 catalytic binding site, with key ligand contacts involving His905 and Tyr884 on SOS1; this positioning produces a steric and/or competitive conflict with the catalytic RAS-binding interface (including an interaction between SOS1 Tyr884 and RAS Arg73), thereby preventing KRAS from engaging SOS1 productively (Hofmann et al., 2021). This pocket-centric mechanism also helps explain SOS1 selectivity—BI-3406 loses activity against SOS2 in biochemical PPI assays, consistent with subtle pocket differences between the paralogs (Hofmann et al., 2021) (Figure 6).

Figure 6 provides structural context for why the SOS1 CDC25 pocket adjacent to the catalytic RAS-binding surface is such an effective intervention point. In the BI-3406 co-crystal structure, the ligand occupies a shallow, hydrophobic cleft on SOS1 that sits immediately next to the RAS^cat^ docking interface (Figure 6A). This placement rationalizes its mechanism as a protein–protein interaction disruptor: BI-3406 engages residues that either participate directly in, or lie immediately adjacent to, the catalytic RAS contact surface (including the Tyr884/His905 region), creating a steric/competitive barrier to productive RAS binding and thereby suppressing SOS-catalyzed nucleotide exchange (Hofmann et al., 2021). Complementing this inhibitor-centric view, the fragment-bound KRAS^G12C^–SOS1^cat^ structure illustrates that the broader RAS-SOS assembly contains ligandable microenvironments at the interface (Figure 6B). The fragment (F1) binds within the complex and is stabilized by a defined network of polar and cation–π interactions, supporting the concept that small molecules can access—and be optimized to exploit—binding sites near the RAS–SOS engagement region to modulate or block complex formation (Hillig et al., 2019).

More recently, orally bioavailable and brain-penetrant SOS1 inhibitors such as MRTX0902 have extended this concept: these agents are best described as selective SOS1 inhibitors that disrupt the SOS1-KRAS protein–protein interaction and have shown pathway suppression, including synergy with KRAS (G12C) allele-specific inhibitors, in preclinical settings (Ketcham et al., 2022). Across these chemotypes, the recurring theme is that the “druggable pocket” is not the flat face of the PPI itself; rather, it is an adjacent cavity on SOS1 that can be occupied by a small molecule to preclude the precise switch-region docking geometry needed for exchange.

### 5.3 What “PPI disruption” means here: blocking productive KRAS–SOS1 complex formation to lower RAS-GTP

In the KRAS–SOS1 context, “PPI disruption” does not typically mean a molecule sits directly between the two proteins like a wedge at a flat interface. Instead, most validated disruptors bind SOS1 at a pocket bordering the catalytic RAS-contact surface, which prevents formation of the nucleotide-exchange-competent complex. Functionally, this blocks SOS1’s ability to catalyze GDP release and thereby reduces the fraction of RAS in the active, GTP-bound state—an effect measurable as decreased cellular RAS-GTP and reduced downstream pathway readouts (e.g., ERK phosphorylation) in KRAS-driven models (Hofmann et al., 2021).

This mechanism is conceptually distinct from (i) allele-specific KRAS covalent inhibitors, which trap mutant KRAS in a particular nucleotide state by binding a KRAS pocket (Cox et al., 2014; Jänne et al., 2022; Skoulidis et al., 2021), and (ii) downstream kinase inhibitors, which suppress signaling despite continued RAS-GTP production. By throttling RAS reloading upstream, KRAS–SOS1 disruptors can blunt the rapid feedback-driven rebound in RAS-GTP that often limits the durability of downstream pathway inhibition and can provide a rationale for vertical combinations (e.g., SOS1 + MEK) and for combinations with KRAS allele-specific inhibitors (e.g., SOS1 + KRAS^G12C^) (Hofmann et al., 2021; Ketcham et al., 2022). Finally, it is important to recognize that SOS1 inhibitors sit at the intersection of structural mechanism and network dynamics: their biochemical endpoint (blocking RAS–SOS1 engagement) maps cleanly to a signaling endpoint (lower RAS-GTP), but the magnitude of pathway suppression will depend on context—RTK/SHP2 drive, SOS2 redundancy, and the degree to which a tumor remains dependent on nucleotide cycling versus mutation-stabilized signaling (Ahmed et al., 2019; Baltanás et al., 2025; Fedele et al., 2021; Hillig et al., 2019; Hofmann et al., 2021; Qian et al., 1998).

## 6 Modalities that block KRAS–SOS1: from peptides to small molecules

### 6.1 Stapled peptides and interface-mimic approaches

Before the emergence of drug-like SOS1 inhibitors, peptide-based interface mimics provided an early experimental demonstration that the KRAS–SOS1 interaction surface could be therapeutically engaged. A prominent example is the development of stabilized α-helices of SOS1 (SAH-SOS1), in which an SOS1-derived helix that normally contacts KRAS was converted into a hydrocarbon-stapled, cell-penetrant peptide (Leshchiner et al., 2015). These peptides bound KRAS in a sequence-specific manner (including wild-type and mutant variants) and inhibited fluorescent nucleotide association *in vitro*. In KRAS-driven cancer cell models, SAH-SOS1 treatment reduced ERK–MAPK pathway phosphorylation and impaired viability, and the authors also demonstrated pathway suppression in a *Drosophila* RAS-driven model (Leshchiner et al., 2015).

Conceptually, SAH-SOS1 and later small molecules both disrupt the RAS:SOS1 interaction, but they do so through different binding modes: SAH-SOS1 acts as a KRAS-directed interface mimic that binds GDP-loaded KRAS in a bimolecular complex, whereas later small molecules engage the RAS:SOS1 interface pocket through a distinct ternary-complex mechanism (Leshchiner et al., 2015). This work taught several durable lessons that informed subsequent efforts: (i) KRAS protein surfaces involved in GEF engagement can be targeted by designed ligands; (ii) in the specific case of KRAS-directed interface mimetics such as SAH-SOS1, functional inhibition can be tracked through direct KRAS biophysical measurements alongside pathway pharmacodynamics (e.g., pERK); and (iii) the KRAS nucleotide cycle can be perturbed by ligands that alter exchange and/or nucleotide association kinetics (Leshchiner et al., 2015).

More specifically, Leshchiner et al. showed that SAH-SOS1A binds recombinant wild-type and mutant KRAS proteins with nanomolar affinity, with fluorescence-polarization measurements in the approximately 100–175 nM range across the KRAS proteins tested. In the biochemical studies, direct inhibition of nucleotide association was demonstrated for wild-type KRAS and KRASG12D in a dose-responsive manner. In the cellular setting, antiproliferative activity was observed across a broader panel of KRAS-driven models harboring KRAS^G12D^, KRAS^G12C,^ KRAS^G12V^, KRAS^G12S^, KRAS^G13D^, and KRAS^Q61H^ mutations, generally with IC_50_ values in the 5–15 μM range. Overall, the available data suggest broad engagement of multiple oncogenic KRAS variants, without strong evidence for major mutant-selective differences in binding affinity, although the cellular potency remained in the micromolar range and likely reflects the additional pharmacologic constraints of the peptide modality. The structural basis for this approach is supported by the KRAS–SOS1 complex structure, in which the interacting SOS1 α-helix contacts KRAS at the exchange interface (PDB ID: 1NVU) (Figure 6) (Leshchiner et al., 2015).

At the same time, the peptide modality highlighted practical constraints for translation. Stapled peptides can achieve high-affinity, high-specificity engagement of extended interfaces, but they frequently face challenges in systemic delivery, endosomal escape, metabolic stability, tissue penetration, and scalable formulation. These limitations motivated a shift toward discovering orally bioavailable small molecules capable of modulating KRAS activation upstream—ideally by targeting SOS1 itself. Subsequent studies continued to explore SOS1-helix–inspired stapled peptides as pan-RAS inhibitors, reinforcing the concept that SOS1-derived interface mimics can engage multiple RAS isoforms and mutant states in cells, albeit with the above modality-specific limitations (Li et al., 2023).

### 6.2 Early small-molecule efforts and assay platforms

In parallel with peptide work, early small-molecule programs sought to inhibit KRAS activation by targeting SOS1’s catalytic machinery directly. Evelyn and colleagues reported a rational design approach combining virtual screening with biochemical validation to identify NSC-658497 as a SOS1 catalytic-site binder (Evelyn et al., 2014). NSC-658497 bound purified SOS1 catalytic domains with micromolar affinity (measured by microscale thermophoresis), competitively disrupted SOS1–RAS binding, and inhibited SOS1-mediated nucleotide exchange in fluorescent GDP-dissociation and GTP-loading assays. ^46^ In cells, the compound reduced growth factor–stimulated RAS activation and attenuated downstream MAPK and PI3K pathway readouts (pERK and pAKT), supporting proof-of-principle that a RAS GEF catalytic interface is chemically tractable (Evelyn et al., 2014).

A subsequent study from the same group systematized these concepts into a scalable discovery workflow, describing a multi-tier screening platform that couples ensemble structure-based virtual screening with high-throughput experimental assays for SOS1 (Evelyn et al., 2015). Using complementary fluorescence-based exchange assays (GDP dissociation and GTP loading) and a large compound collection, the authors identified two chemically distinct inhibitor series that bound SOS1 with micromolar affinity, inhibited SOS1 catalytic activity *in vitro*, and disrupted SOS1–RAS interactions (Evelyn et al., 2015). Mutagenesis and structure–activity relationship (SAR) analyses suggested that the hits occupied distinct positions within the SOS1 pocket(s) relevant to RAS engagement, providing early evidence that more than one site could be exploited to suppress exchange-factor function (Evelyn et al., 2015).

Although these early compounds were generally micromolar and not yet optimized for the exposure, selectivity, and physicochemical properties required for *in vivo* use, they were pivotal in establishing key enabling infrastructure: robust nucleotide-exchange assays suitable for miniaturization, orthogonal binding/competition experiments to confirm target engagement, and structural hypotheses for how small molecules could interfere with SOS1–RAS complex formation (Evelyn et al., 2014; Evelyn et al., 2015). These capabilities directly anticipated the later “modern era” of potent, nanomolar SOS1 binders discovered by fragment and structure-guided approaches, which ultimately yielded translational-quality SOS1 inhibitors and clinical candidates.

## 7 Medicinal chemistry evolution of SOS1 PPI disruptors

### 7.1 Tool compound era to BAY-293

The first wave of SOS1-targeted chemistry established two critical principles for the field: (i) the catalytic domain of SOS1 contains a ligandable pocket that can be engaged by small molecules, and (ii) occupancy of this pocket can translate into functional disruption of the KRAS-SOS1 complex, reducing RAS-GTP formation and downstream signaling. A defining milestone was the report of quinazoline-based inhibitors that directly block the SOS1-KRAS interaction, culminating in the chemical probe BAY-293 (Hillig et al., 2019) (Figure 4).

**FIGURE 7:**
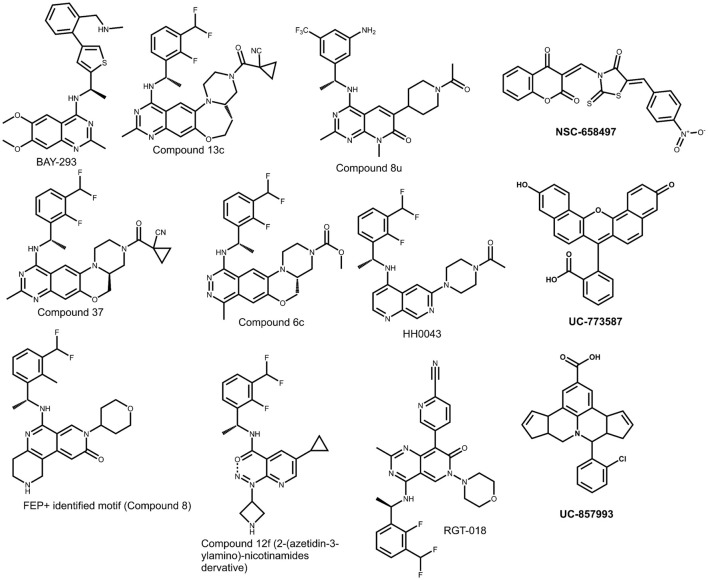
Representative small-molecule scaffolds highlighting recent medicinal-chemistry optimization trends in the SOS1–KRAS axis.

In biochemical assays, BAY-293 (Figure 7) inhibited the SOS1-KRAS interaction with nanomolar potency (reported IC_50_ = 21 nM) and reduced RAS pathway output in KRAS-dependent cells, making it the first broadly adopted benchmark for target engagement and pathway pharmacology in the SOS1 space (Hillig et al., 2019). From a medicinal chemistry standpoint, BAY-293 did not yet meet clinical-candidate standards because, although it was an excellent cell-active chemical probe, it was not advanced with the integrated exposure, physicochemical, selectivity, and broader developability profile typically required for *in vivo* translation and clinical development. Nevertheless, it set the blueprint for subsequent optimization: maximizing affinity for the SOS1 pocket while preserving cell permeability, and de-risking selectivity liabilities that can accompany kinase-like heteroaromatic cores (a recurring theme in later SOS1 optimization studies), while confirming that biochemical PPI disruption correlates with decreased RAS-GTP and reduced ERK signaling in cells (Hillig et al., 2019; Ramharter et al., 2021) (Figure 4).

### 7.2 From potent probe to translational pharmacology: BI-3406

Boehringer Ingelheim advanced the concept from a probe to a more translationally oriented inhibitor with BI-3406 (Figure 3), which binds the same SOS1 pocket and disrupts productive SOS1–KRAS engagement. In the discovery study, an earlier quinazoline hit (BI-68BS) showed SOS1 binding with K_D_ = 470 nM and disrupted the SOS1–KRAS interaction with IC_50_ = 1.3 μM; subsequent structure-guided optimization yielded BI-3406 with substantially improved activity. In biochemical protein–protein interaction assays, BI-3406 potently disrupted SOS1 interaction with both KRAS^G12C^ and KRAS^G12D^, while losing activity when SOS1 was replaced by its paralog SOS2, consistent with strong SOS1 selectivity. The medicinal chemistry campaign also introduced a 2-methyl substituent specifically to eliminate residual kinase activity from the quinazoline core, and this was evaluated in a 324-kinase panel during optimization (Hofmann et al., 2021).

In cells, BI-3406 inhibited RAS-GTP with IC_50_ values of 83–231 nM in SOS1/KRAS-dependent models such as NCI-H358 (KRAS^G12C^) and A549 (KRAS^G12S^), and reduced pERK across RAS-mutant cell lines with IC_50_ values of 17–57 nM. In a broader 3D proliferation panel of 40 cancer cell lines, sensitivity was summarized using an IC_50_ cutoff of 100 nM, with preferential activity in KRAS-pathway-dependent models. These data provided a sharper demonstration of how upstream control can be exploited to manage pathway adaptation: by suppressing SOS1-mediated nucleotide exchange, BI-3406 lowers the pool of KRAS–GTP that fuels MAPK signaling and limits the capacity for rapid rebound activation (Hofmann et al., 2021).

The translational relevance of BI-3406 was supported by oral *in vivo* studies. In MIA PaCa-2 xenografts, 50 mg/kg twice daily produced pathway modulation for up to 10 h, whereas pERK returned toward baseline by 24 h as compound exposure declined. In the same model, 12 or 50 mg/kg twice daily was well tolerated and produced dose-dependent tumor growth inhibition, and 50 mg/kg twice daily also showed activity across additional xenografts including SW620 (KRAS^G12V^), LoVo (KRAS^G13D^), and A549 (KRAS^G12S^). Importantly, combining BI-3406 (50 mg/kg twice daily) with trametinib (0.1–0.125 mg/kg twice daily) produced substantial tumor regressions in the MIA PaCa-2 model, supporting the “feedback-control” rationale for SOS1/MEK cotargeting (Hofmann et al., 2021).

### 7.3 Clinical-candidate-quality chemistry: MRTX0902 and design goals

Mirati’s MRTX0902 exemplifies the next medicinal chemistry leap: maintaining potent SOS1 pocket binding and KRAS–SOS1 PPI disruption while explicitly engineering properties needed for systemic dosing, including oral exposure and central nervous system penetration. Quantitatively, MRTX0902 showed SOS1 binding K_i_ = 2 nM and a representative cellular IC_50_ = 29 nM in MKN1 cells. Its selectivity profile was also reported explicitly: SOS2 KRAS^WT^ GDP-exchange IC_50_ > 10,000 nM and EGFR IC_50_ > 10,000 nM. In addition, across a 78-target safety panel, 74 targets showed EC/IC_50_ > 10 μM, supporting a relatively clean off-target profile. These numerical data make clear that the campaign did not merely produce a potent SOS1 binder, but one with a deliberately widened selectivity margin over both the paralog SOS2 and kinase liabilities such as EGFR (Ketcham et al., 2022).

Drug-like disposition was also quantified. *In vitro*, MRTX0902 showed Caco-2 P_app (A-to-B) = 32.3 × 10^−6^ cm/s with an efflux ratio of 1.5, consistent with good permeability and limited efflux. Across species, intravenous pharmacokinetics showed low clearance (4.4 mL/min/kg in mouse, 14.6 in rat, 7.6 in dog), low steady-state volume of distribution (0.28, 0.28, and 0.48 L/kg, respectively), and short IV half-life (1.3, 0.62, and 0.86 h). Oral dosing yielded bioavailability values of 69% in mouse, 83% in rat, and 38% in dog, which provides the quantitative basis for describing the compound as orally bioavailable (Daley et al., 2025; Ketcham et al., 2022).

The brain-penetration claim can likewise be stated numerically. After a 100 mg/kg oral dose in mice, mean CSF concentrations were 209 nM at 1 h and 36 nM at 8 h, with CSF:free plasma (K_p_,uu) values of 1.56 and 1.03, respectively. These CSF exposures exceeded both the reported MKN1 cellular IC_50_ (29 nM) and the calculated efficacious free C_avg_ of 25 nM for up to 8 h, supporting the designation of MRTX0902 as brain-penetrant. *In vivo*, the compound also showed single-agent efficacy, producing 41% and 53% tumor growth inhibition at 25 and 50 mg/kg twice daily, respectively, in the MIA PaCa-2 xenograft model.

Pharmacologically, MRTX0902 is positioned as combination-ready. In the later mechanistic study, MRTX0902 inhibited pERK with IC_50_ < 100 nM in 16 KRAS–MAPK pathway-mutant cell lines and showed antiproliferative activity with IC_50_ < 250 nM in 13 of 20 tested lines, including models with EGFR, PTPN11, SOS1, NF1, KRAS, and class III BRAF alterations. *In vivo*, oral MRTX0902 at 25 or 50 mg/kg twice daily in NCI-H1435 (NF1-mutant) xenografts produced 50% and 73% tumor growth inhibition, respectively, with pharmacodynamic suppression of pERK tracking with plasma exposure. Together, these numerical data better define the potency, selectivity, oral exposure, and translational profile of MRTX0902 beyond a purely qualitative description (Sudhakar et al., 2024) (Figure 4).

### 7.4 New scaffolds and optimization trends (2022–2025 landscape)

From 2022 onward, the literature reflects rapid scaffold diversification around the same fundamental binding site, alongside consistent optimization pressures: (i) potency versus permeability (and solubility), (ii) selectivity versus kinases and other off-targets, (iii) selectivity for SOS1 over SOS2, (iv) exposure and half-life supporting sustained target coverage, and (v) combination performance (especially with MEK inhibitors and KRAS allele-specific drugs).

Tetracyclic quinazoline refinements (compound 13c, Figure 7). He and colleagues reported an orally bioavailable series, identifying compound 13c with strong biochemical and cellular activity (reported biochemical IC_50_ = 3.9 nM; cellular IC_50_ = 21 nM in a pERK assay) and favorable oral exposure (reported bioavailability 86.8% in beagle dogs). In a MIA PaCa-2 xenograft model, oral dosing was reported to suppress tumor growth by 83%, supporting the idea that high-affinity SOS1 pocket engagement can translate into *in vivo* efficacy when paired with adequate exposure (He et al., 2022).

Efficacy-improving quinazoline derivatives (compound 37, Figure 7). A separate optimization effort disclosed a tetracyclic quinazoline scaffold with improved pharmacokinetics, where compound 37 showed higher oral exposure and longer half-life than BI-3406 in mice, along with 71% tumor growth inhibition in a Mia-PaCa-2 xenograft model. Notably, the authors also reported limited CYP and hERG liabilities in preclinical assays, reflecting the field’s emphasis on de-risking safety while preserving potency (Zhang et al., 2022).

Pyrido [2,3-d]pyrimidin-7-one scaffold (compound 8u, Figure 7). Liu and colleagues introduced a distinct core and identified compound 8u with activities comparable to BI-3406 in biochemical and 3D cell growth assays. The compound inhibited ERK and AKT signaling in KRAS-mutant pancreatic cancer models (MIA PaCa-2 and AsPC-1) and showed synergistic antiproliferative effects when combined with KRAS (G12C) or KRAS (G12D) inhibitors, underscoring the recurring “combination-ready” logic in SOS1 medicinal chemistry (Liu et al., 2023) (Figure 6).

Tetracyclic phthalazine derivatives (compound 6c, Figure 7). Building on the concept that phthalazine-based cores can support potent SOS1 binding while reducing EGFR-related liabilities, a reported series identified compound 6c with favorable oral bioavailability (reported 65.8%) and antitumor efficacy in a Mia-PaCa-2 xenograft model; the same study noted safety concerns for the compound at high dosage, emphasizing the importance of margin and tolerability alongside potency (He et al., 2023) (Figure 6).

1,7-Naphthyridine “scaffold-hopping” program (HH0043; Figure 7). A scaffold-hopping strategy generated novel 1,7-naphthyridine inhibitors, with compound 10f (HH0043) showing potent biochemical and cellular activity and favorable pharmacokinetics. Oral HH0043 produced 76% tumor growth inhibition in a KRAS (G12C)-mutant NCI-H358 xenograft model, and was reported to outperform BI-3406 at the same dose (TGI 76% vs. 49%) (Li D. et al., 2024) (Figure 6).

Xiao and colleagues described RGT-018 (Figure 7) as a potent and selective SOS1 inhibitor with “single-digit nanomolar” biochemical potency for blocking KRAS:SOS1 interaction and high selectivity against SOS2. *In vitro*, RGT-018 inhibited KRAS signaling and proliferation across a broad spectrum of KRAS-driven cancer cell lines; combinations with MEK inhibitors or KRAS (G12C) inhibitors were reported to produce tumor regressions in xenograft models, and the authors further reported activity against resistance associated with clinically acquired KRAS mutations (Xiao et al., 2024) (Figure 6).

Computation-guided salt-bridge exploitation (2025). Leffler and colleagues used free-energy perturbation simulations to guide the design of SOS1 inhibitors that explore interactions with an acidic region on the perimeter of the binding pocket. In that series, introduction of basic substituents near SOS1 residues E906/E909 was reported to be associated with substantial affinity gains, and the authors proposed that solvent-exposed electrostatic interactions contributed to this effect. However, in a review context, this result should be interpreted cautiously, as the observed potency improvements may reflect not only the proposed salt-bridge interaction but also broader structure–activity and physicochemical consequences of the modifications (compound 8, Figure 7) (Leffler et al., 2025). Nicotinamide derivatives engaging an acidic SOS1 region (2025). A distinct class of 2-(azetidin-3-ylamino)-nicotinamides (compound 12f, Figure 7) was reported to achieve potent biochemical activity with minimal hERG inhibition, using a binding concept that positions basic substituents toward an acidic SOS1 region. Although the reported pharmacokinetic profile remained suboptimal, the work highlights continued exploration of alternative binding modes to broaden the chemical space of SOS1 inhibitors (Li et al., 2025).

#### Clinical-stage positioning

Translationally, the most mature SOS1 programs have converged on combination strategies. BI-1701963 (derived from the BI-3406 program) has been evaluated clinically as monotherapy and in combination with the MEK inhibitor trametinib in KRAS-mutant solid tumors, and has also been tested in combination with adagrasib (MRTX849) in KRAS (G12C)-mutant cancers (Haddad et al., 2025). MRTX0902 is in clinical evaluation in solid tumors, and ongoing trial designs emphasize combination-readiness (including combinations that pair SOS1 inhibition with pathway blockade or KRAS allele-specific inhibition). More recently, BAY3498264 has entered clinical evaluation, including a study combining BAY3498264 with sotorasib in KRAS (G12C)-mutant solid tumors (Guthof et al., 2025; Malhotra et al., 2024; Sudhakar et al., 2024).

Across these programs, medicinal chemistry has steadily shifted from “can we bind SOS1 and disrupt the KRAS-SOS1 interaction?” to “can we deliver durable target coverage in patients without dose-limiting liabilities?” The recurring design priorities are (i) high-affinity pocket binding coupled to demonstrable suppression of RAS-GTP, (ii) clean selectivity (particularly avoiding kinase off-targets), (iii) oral pharmacokinetics compatible with chronic dosing, and (iv) rational pairing with complementary inhibitors to prevent or delay adaptive signaling and resistance (Zhao et al., 2017). These themes set the stage for the next chapter of the field: optimizing patient-relevant exposure and defining the combination regimens most likely to deliver durable clinical benefit.

## 8 Pharmacology: what SOS1 inhibition does in cells and tumors

### 8.1 Primary pharmacodynamic readouts: RAS-GTP reduction, pERK suppression, and adaptive rebound

Across SOS1 inhibitor series, the most direct cellular pharmacodynamic (PD) marker is a decrease in the abundance of active, GTP-loaded RAS. In practice, this is most often quantified using RAF-RBD pull-down assays (or related affinity capture formats) followed by immunoblotting for KRAS/NRAS/HRAS, enabling measurement of total RAS-GTP or isoform-resolved pools (Evelyn et al., 2014; Evelyn et al., 2015; Hillig et al., 2019; Hofmann et al., 2021; Sudhakar et al., 2024). Downstream, reduced RAS-GTP typically translates into suppression of MAPK pathway signaling, most commonly tracked by decreased ERK phosphorylation (pERK) and, in some studies, reduced expression of ERK-regulated transcriptional targets such as DUSP-family phosphatases (Hillig et al., 2019; Hofmann et al., 2021; Sudhakar et al., 2024).

A second hallmark of SOS1 pharmacology is its impact on adaptive rebound programs that follow distal pathway inhibition. MEK inhibition can relieve ERK-dependent negative feedback, driving RTK reactivation and restoration of upstream RAS signaling (Kitai et al., 2016; Kolch et al., 2023). In KRAS-driven models, this rebound can be observed as partial recovery of pERK (and/or upstream signaling) despite continued drug exposure, and it is frequently accompanied by increased RTK and adaptor signaling (Kitai et al., 2016). BI-3406 provided a clear pharmacologic illustration that suppressing RAS re-loading upstream can blunt this rebound: SOS1 inhibition reduced RAS-GTP formation and improved the depth and durability of MAPK suppression when combined with MEK inhibition, translating into stronger antitumor activity than either agent alone in preclinical KRAS-driven models (Hofmann et al., 2021).

This “re-loading control” concept also applies to direct KRAS inhibitors whose binding preferences depend on nucleotide state. Covalent KRASG12C inhibitors bind the GDP-bound (inactive) conformation, and treatment can trigger feedback activation of wild-type RAS that constrains efficacy (Isermann et al., 2025; Ostrem and Shokat, 2016; Ryan et al., 2022). In this setting, SOS1 inhibition can reduce the upstream exchange activity that replenishes RAS-GTP pools and has been reported to enhance KRASG12C inhibitor efficacy and delay the emergence of resistance in lung adenocarcinoma models (Daley et al., 2025). Consistent with this framework, the SOS1 PPI disruptor MRTX0902 demonstrated pathway suppression and synergy with KRAS and MEK inhibitors in preclinical studies, supporting combination-oriented development rather than reliance on monotherapy PD endpoints alone (Ketcham et al., 2022; Sudhakar et al., 2024).

### 8.2 Context dependence: KRAS allele, RTK activity, and baseline pathway tone

The magnitude of SOS1 inhibitor responses varies across models, reflecting both allele-specific biochemistry and the signaling context that controls nucleotide exchange. Different KRAS mutants differ in intrinsic hydrolysis, GAP sensitivity, and the fraction of time spent in GDP-bound states, which can alter how strongly upstream exchange factors influence steady-state RAS-GTP (Isermann et al., 2025; Kolch et al., 2023; Ostrem and Shokat, 2016). As a result, tumors driven by alleles with substantial ongoing cycling—and especially those with high RTK input—may remain more sensitive to upstream modulation of exchange than contexts where mutant KRAS is effectively insulated from upstream regulation (Isermann et al., 2025; Kolch et al., 2023).

RTK activity is a dominant determinant of SOS1 inhibitor pharmacology because RTKs control SOS1 membrane recruitment and activation via adaptor proteins. MEK inhibition–induced feedback can further amplify RTK signaling, making the “baseline pathway tone” and feedback susceptibility of a tumor central to predicting response (Hofmann et al., 2021; Kitai et al., 2016; Kolch et al., 2023). Experimental studies illustrate this principle in multiple ways: (i) MEK inhibition can produce RTK-driven rebound that is context dependent (including cell state effects such as epithelial-to-mesenchymal transition (EMT)), and (ii) proximal pathway interventions that limit upstream signaling can deepen MAPK suppression and enhance the effect of both distal kinase inhibitors and nucleotide-state-selective KRAS inhibitors (Fedele et al., 2021; Kitai et al., 2016; Ryan et al., 2022).

Finally, the distribution of active RAS among mutant KRAS and co-existing wild-type RAS isoforms influences both biomarker interpretation and therapeutic logic. For example, KRASG12C inhibitor treatment can drive compensatory activation of wild-type RAS, which can sustain downstream signaling even when the mutant protein is inhibited (Ryan et al., 2022). Because SOS1 is an upstream exchange factor for multiple RAS isoforms, SOS1 inhibition provides a plausible route to reduce both mutant- and wild-type-driven RAS-GTP pools in RTK-high settings, potentially explaining why SOS1 inhibitors frequently show their greatest impact in combination regimens that confront feedback and bypass signaling (Daley et al., 2025; Hofmann et al., 2021; Isermann et al., 2025).

An important implication of SOS1 blockade is that its pharmacology is not restricted to mutant KRAS alone. Because SOS1 catalyzes nucleotide exchange across Ras isoforms, and because pharmacodynamic studies commonly monitor KRAS/NRAS/HRAS-GTP pools, inhibition of SOS1 may also attenuate signaling through wild-type NRAS and HRAS, particularly in RTK-high settings where paralog compensation contributes to pathway rebound. This broader isoform coverage may be therapeutically advantageous by limiting wild-type RAS-mediated adaptive rewiring during KRAS-directed therapy, but it also reinforces that the magnitude of SOS1 inhibitor benefit will depend on context, especially RTK input and the relative contribution of co-existing wild-type Ras paralogs to downstream signaling (Daley et al., 2025; Hillig et al., 2019; Hofmann et al., 2021; Ryan et al., 2022; Sudhakar et al., 2024).

### 8.3 On-target validation strategies: biochemical disruption, structural biology, and cellular engagement

Because SOS1 inhibitors are designed to disrupt a protein–protein interaction rather than inhibit a classical enzymatic active site, rigorous on-target validation has been a defining feature of the field. Leading programs have generally converged on three complementary evidence layers. First, direct biochemical binding and functional inhibition are established using purified proteins, including biophysical assays for direct SOS1 engagement, recombinant-protein assays that measure disruption of the SOS1:RAS interaction (e.g., FRET- or HTRF-based PPI assays), and nucleotide-exchange functional assays that quantify inhibition of SOS1-catalyzed GDP release and/or GTP loading (Evelyn et al., 2014; Evelyn et al., 2015; Hillig et al., 2019; Hofmann et al., 2021; Ketcham et al., 2022).

Second, structural biology provides high-confidence mechanistic validation by showing compounds bound within the SOS1 pocket adjacent to the KRAS-binding interface and rationalizing how ligand occupancy disrupts productive complex formation. Co-crystal structures were central to the development of BAY-293 and BI-3406, and structure-guided iterations underpinned later candidate-quality series (Hillig et al., 2019; Hofmann et al., 2021; Ketcham et al., 2022). Structure also enables informative resistance and specificity tests, such as engineering SOS1 pocket mutations predicted to reduce ligand affinity and assessing whether those changes attenuate cellular PD responses (Hofmann et al., 2021; Ketcham et al., 2022).

Third, cellular engagement and pathway modulation are linked using orthogonal assays that connect target engagement to downstream consequences. Across papers, this can include direct cellular target-engagement formats—such as NanoBiT- or other complementation-based assays that monitor disruption of the RAS:SOS1 interaction in cells—together with dose-dependent suppression of RAS-GTP, reduced pERK (and sometimes pAKT), and demonstration that SOS1 pharmacology reshapes adaptive feedback behavior when combined with MEK/ERK or direct KRAS inhibitors (Daley et al., 2025; Hillig et al., 2019; Hofmann et al., 2021; Ryan et al., 2022; Sudhakar et al., 2024). Where possible, investigators strengthen on-target claims with genetic perturbations (SOS1 knockdown/knockout phenocopy), rescue strategies (re-expression of SOS1 variants), or use of close inactive analogs as negative controls—approaches that help distinguish direct SOS1 biology from indirect effects on RTKs or other upstream regulators (Baltanás et al., 2025; Evelyn et al., 2014; Evelyn et al., 2015; Hofmann et al., 2021).

## 9 Combination strategies: why SOS1 inhibitors are “multiplier” drugs

A recurring theme across SOS1 programs is that these agents rarely act as simple “standalone” pathway blockers. Because SOS1 sits at a proximal choke point for GDP-to-GTP exchange across RAS isoforms, inhibiting SOS1 can lower the RAS-GTP pool that feeds multiple effectors, blunt adaptive reactivation driven by receptor tyrosine kinases (RTKs), and increase the depth and durability of inhibition achieved by downstream or allele-specific drugs (Hofmann et al., 2021; Kitai et al., 2016). This “multiplier” behavior is most evident in combinations. Here, we frame the rationale and evidence in two buckets: (i) vertical inhibition (multiple nodes along the RTK–SOS–RAS–RAF/MEK/ERK axis) and (ii) parallel escape control (pairing MAPK suppression with blockade of alternative survival pathways such as PI3K/AKT/mTOR).

### 9.1 With MEK inhibitors: feedback control as the rationale

MEK inhibitors can produce rapid MAPK pathway suppression, but their clinical impact in KRAS-mutant tumors has been limited by both intrinsic and adaptive resistance. A key mechanistic driver of adaptation is relief of ERK-dependent negative feedback, which can upregulate RTK signaling and re-establish RAS-GTP and downstream phosphorylation, particularly in contexts prone to RTK feedback activation (for example, EMT-associated states in KRAS-mutant lung cancer) (Blumenschein et al., 2015; Kitai et al., 2016). By targeting nucleotide loading upstream, SOS1 inhibition is well positioned to suppress this rebound. In KRAS-driven cell and xenograft models, the SOS1 inhibitor BI-3406 deepened and prolonged MAPK pathway suppression when combined with trametinib and produced superior antitumor activity compared with either agent alone; this combination logic was a central rationale for advancing SOS1 inhibitors into MEK-inhibitor combinations in early clinical testing (Hofmann et al., 2021; Sütcüoğlu et al., 2025).

More recently, genotype-stratified work has clarified when the MEK + SOS1 strategy is most effective. In 3D spheroid models of KRAS-mutant lung and colorectal adenocarcinoma, BI-3406 synergized with trametinib and prevented emergence of trametinib-resistant spheroid-initiating cell populations in KRAS G12/G13 models without PIK3CA comutations, whereas KRASQ61 and/or PIK3CA-mutant contexts showed reduced sensitivity to the combination. These results reinforce that SOS1 inhibition can be a robust feedback-control partner for MEK inhibition, but that the magnitude of benefit depends on KRAS allele and co-mutation landscape (Daley et al., 2023).

### 9.2 With KRAS G12C inhibitors: blocking reloading and adaptive rewiring

**FIGURE 8:**
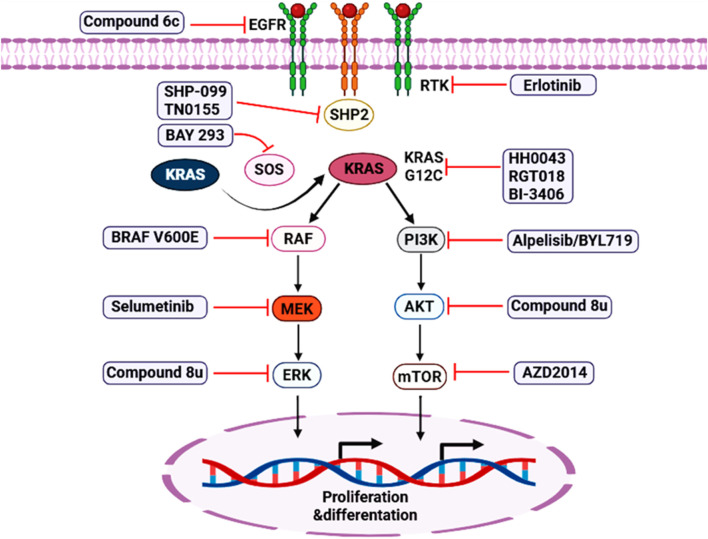
Targeted therapy nodes in RTK–SHP2–SOS–KRAS signaling and downstream effector pathways. Schematic overview of representative targeted agents mapped onto the EGFR/RTK→SHP2/SOS→KRAS axis and its major downstream branches, including the RAF–MEK–ERK and PI3K–AKT–mTOR cascades that drive tumor cell proliferation and differentiation. Inhibitor classes shown include EGFR/RTK inhibitors (e.g., erlotinib and compound 6c), SHP2 inhibitors (e.g., SHP-099 and TN0155), SOS1 inhibitors (e.g., BAY-293), KRAS-directed inhibitors (e.g., HH0043, RGT018, BI-3406), and downstream RAF/MEK/ERK and PI3K/AKT/mTOR pathway inhibitors (e.g., selumetinib, alpelisib/BYL719, and AZD 2014). Red inhibitory symbols indicate the point of pathway blockade.

Covalent KRAS (G12C) inhibitors bind the GDP state of the mutant protein, and their pharmacology is therefore tightly coupled to nucleotide cycling. Upstream signaling that promotes GTP loading (including SOS1-mediated exchange) can reduce the fraction of drug-bindable KRAS (G12C)-GDP and can also drive compensatory activation of wild-type RAS isoforms. A proximal SOS1 inhibitor can counter both processes by shifting the nucleotide balance toward GDP and by limiting the overall RAS-GTP pool that sustains pathway output (Daley et al., 2025; Ostrem and Shokat, 2016; Ryan et al., 2022). *In vivo* evidence for this synergy is now strong. In KRASG12C xenograft and patient-derived models, BI-3406 combined with the KRAS (G12C) inhibitor adagrasib produced deeper tumor regressions than adagrasib alone across multiple NSCLC and colorectal cancer models; responses were broadly comparable to adagrasib combined with either an SHP2 inhibitor (for example, TNO155/SHP099) or an EGFR-directed antibody (cetuximab) in the same study. Mechanistically, BI-3406 co-treatment further reduced total RAS-GTP (including wild-type RAS isoforms and muscle RAS oncogene homolog (MRAS)), attenuated MAPK pathway activity rebound at later timepoints in selected models, and suppressed the outgrowth of resistant clones; importantly, KRAS (G12C) engagement (KRAS “shift”) remained evident, consistent with preserved covalent target binding (Thatikonda et al., 2024) (Figure 8).

This proximal “reloading blockade” concept also aligns with the preclinical positioning of newer SOS1 inhibitors. For example, MRTX0902 was explicitly optimized as an orally bioavailable, combination-ready SOS1 pocket ligand, and preclinical models show enhanced pathway suppression and antitumor activity when SOS1 inhibitors are paired with KRAS (G12C) inhibitors (Ketcham et al., 2022; Sudhakar et al., 2024; Thatikonda et al., 2024).

### 9.3 With RTK/SHP2 and other pathway nodes: vertical inhibition versus parallel escape

Beyond MEK and KRAS (G12C), combination strategies with SOS1 inhibitors fall into two mechanistic classes. Vertical inhibition pairs SOS1 blockade with other nodes in the same signaling cascade (RTKs, SHP2, RAF/MEK/ERK, or allele-specific RAS inhibitors) to reduce pathway flux and suppress feedback-driven rebound. Parallel escape control pairs SOS1/MAPK suppression with blockade of alternative survival circuits (most commonly PI3K/AKT/mTOR) that can sustain growth when MAPK output is constrained. Vertical inhibition with RTK-directed therapy is supported by several lines of evidence. In EGFR-mutant lung adenocarcinoma models, the SOS1 inhibitor BAY-293 showed marked synergy with EGFR tyrosine kinase inhibitors (notably osimertinib) under 3D spheroid conditions, with combined treatment more effectively suppressing both ERK and AKT pathway phosphorylation than either agent alone - consistent with SOS1 functioning as a convergent mediator of RTK input (Hillig et al., 2019; Theard et al., 2020).

In KRASG12C-driven models, vertical combinations that converge on the RTK–SHP2–SOS1 module appear partially interchangeable: adagrasib plus BI-3406 achieved antitumor activity comparable to adagrasib plus SHP2 inhibition or EGFR blockade in matched xenograft studies, and transcriptomic signatures induced by KRAS (G12C) inhibitor + BI-3406 overlapped substantially with those induced by KRAS (G12C) inhibitor + SHP2 inhibition. These results suggest that the dominant benefit can arise from limiting upstream RAS loading, even when the specific upstream node differs (Fedele et al., 2021; Thatikonda et al., 2024).

Parallel escape control is increasingly motivated by the observation that tumors can maintain survival through PI3K/AKT/mTOR signaling or other bypass pathways during sustained MAPK suppression. In the same KRASG12C combination study, high-throughput combination screening highlighted additivity with PI3K pathway inhibition (for example, alpelisib/BYL719), and resistant or nonresponsive contexts showed enrichment of signatures linked to RTK and PI3K pathway activity. Such findings support a framework in which SOS1 inhibitors provide a strong vertical partner for MAPK-directed therapy, while PI3K/AKT/mTOR or other escape nodes may be rational second partners in selected molecular or transcriptional contexts (Thatikonda et al., 2024) (Figure 8).

## 10 Resistance and durability: expected escape routes for SOS1 blockade

SOS1 inhibitors aim to blunt the ability of receptor tyrosine kinase (RTK) signaling to load RAS with GTP, thereby suppressing downstream MAPK output and reducing the “reloading” that undermines many targeted therapies. As with other proximal pathway interventions, the dominant durability challenge is not the loss of an initial pharmacodynamic effect, but rather the emergence of compensatory circuitry that restores RAS-MAPK flux or shifts dependence to parallel survival programs (Riedl et al., 2025; Sheffels and Kortum, 2021; Thatikonda et al., 2024).

### 10.1 Upstream bypass: RTKs, SHP2, and alternative exchange capacity

A straightforward escape route for SOS1 blockade is to increase upstream drive. In multiple RAS contexts, RTK feedback can rapidly restore pathway output through heightened receptor signaling and activation of wild-type RAS pools. In KRASG12C colorectal cancer models, EGFR signaling is a major brake on durable MAPK suppression: KRASG12C inhibitors can trigger rapid EGFR-dependent reactivation of ERK, and EGFR blockade restores deeper and more durable suppression. This behavior highlights an important general principle for SOS1 inhibitors: tumors may evade reduced nucleotide exchange by amplifying receptor signaling strength (via receptor upregulation, ligand production, or altered adaptor usage), thereby preserving enough RAS-GTP to maintain downstream signaling (Amodio et al., 2020; Ryan et al., 2022).

SHP2 sits at the RTK–GRB2–SOS axis and helps couple a range of receptors to RAS activation. In RAS-driven models, SHP2 inhibition can prevent or delay adaptive rewiring that otherwise restores pathway signaling under downstream MAPK inhibition, providing a mechanistic rationale for combining SOS1 inhibition with SHP2 or RTK blockade in settings where receptor feedback is prominent and heterogeneous across receptors (Fedele et al., 2021; Jänne et al., 2022).

A second upstream bypass is “exchange capacity” compensation via alternative RAS GEFs. Although SOS1 is the dominant RTK-coupled GEF in many epithelial contexts, SOS2 can be essential in specific transformation settings and can tune the threshold of EGFR-dependent signaling. Beyond SOS2, other RasGEFs such as members of the RasGRP family may also provide alternative exchange capacity in lineage-restricted contexts. We therefore now note that resistance to SOS1 blockade may not be limited to SOS2 compensation alone, but could also emerge through engagement of other Ras activators that sustain RAS-MAPK flux despite reduced SOS1-dependent exchange (Lambert and Reuther, 2006). Recent work in EGFR-mutant NSCLC indicates that SOS2-dependent signaling can shape resistance programs to EGFR kinase inhibitors, including adaptive engagement of PI3K/AKT nodes, implying that selective pressure on SOS1 may favor SOS2-mediated compensation in some contexts. This has helped motivate emerging medicinal chemistry programs aimed at SOS2 binders as potential future complements to SOS1 inhibition (Sheffels et al., 2018; Theard et al., 2020; Zak et al., 2025).

### 10.2 Downstream bypass: reactivating MAPK/ERK or switching to parallel survival pathways

Downstream bypass mirrors what has been learned from KRASG12C inhibitor resistance: tumors can restore RAS-MAPK output through convergent alterations across the cascade. Clinical and cfDNA-based analyses have documented polyclonal resistance patterns that include new KRAS alterations (including switch-II pocket mutations), KRAS amplification, and secondary events in NRAS or HRAS, RAF/BRAF alterations, and MAP2K1 changes, frequently co-occurring within the same patient. In colorectal cancer treated with combined KRASG12C and EGFR blockade, acquired resistance similarly converges on MAPK reactivation alongside additional RTK and pathway alterations, reinforcing that pathway reactivation can emerge at multiple levels in parallel (Riedl et al., 2025; Tanaka et al., 2021; Yaeger et al., 2023) (Figure 8).

These datasets are directly relevant to SOS1 blockade for two reasons. First, they define the spectrum of alterations that can re-establish ERK flux even when upstream nucleotide loading is constrained. Second, they highlight where SOS1 inhibition might retain or even gain value: *in vitro* studies of acquired resistance to KRASG12C inhibitors cataloged multiple second-site KRAS mutations and showed that constraining upstream RAS-GTP formation (with SOS1 inhibition) together with MEK blockade can suppress signaling and growth in select resistant contexts. This suggests SOS1 inhibitors may function as a “backstop” for MAPK control in combination regimens, including scenarios where on-target KRAS mutations undermine KRASG12C inhibitors (Koga et al., 2021; Thatikonda et al., 2024).

Direct on-target resistance to SOS1 inhibitors remains largely theoretical at present. Because most modern compounds bind a defined pocket on SOS1, amino-acid substitutions that weaken ligand binding or alter autoinhibitory dynamics could, in principle, blunt drug action, analogous to other allosteric targeted therapies. As clinical experience with SOS1 inhibitors expands, monitoring for SOS1 mutations and for compensatory changes in SOS1/SOS2 expression may be informative when resistance emerges (Riedl et al., 2025; Sheffels and Kortum, 2021).

Parallel-pathway switching is another common route. In KRASG12C systems, adaptive resistance can involve enhanced adhesion and integrin signaling and increased PI3K/AKT engagement, supporting survival despite incomplete MAPK suppression. Conceptually, the same biology is expected to erode the depth of response to SOS1 inhibitors, especially in lineages where PI3K/AKT tone is high at baseline or readily inducible by microenvironmental cues (Kitai et al., 2016; Mohanty et al., 2023).

### 10.3 Lineage/state switching and microenvironmental signaling

Cell state plasticity can reduce dependence on the specific wiring that SOS1 inhibitors disrupt. Epithelial-to-mesenchymal transition (EMT) and hybrid E/M states are repeatedly linked to reduced sensitivity to MAPK pathway suppression and increased reliance on alternative RTKs and survival programs. In KRAS-mutant models, EMT has been associated with resistance to MEK inhibition and a compensatory dependence on fibroblast growth factor receptor (FGFR) signaling, exemplifying how a lineage shift can re-route upstream inputs toward alternative RTKs and non-MAPK survival pathways and reduce reliance on canonical RTK–SOS1–RAS coupling (Kitai et al., 2016).

The tumor microenvironment can further amplify bypass by supplying ligands (EGF-family, HGF, FGFs) that intensify RTK signaling or by activating stromal pathways that feed PI3K/AKT and other pro-survival modules. Because SOS1 inhibitors mainly dampen signaling throughput rather than abolish it, ligand-rich contexts may demand vertical combinations (RTK or SHP2 plus SOS1) to prevent rebound and sustain pathway suppression (Sheffels and Kortum, 2021; Theard et al., 2020).

### 10.4 Biomarkers: Who benefits, who escapes quickly?

Mechanistically grounded biomarkers for SOS1 inhibitors are likely to fall into two buckets: (i) markers of upstream dependence where KRAS output remains coupled to RTK–GRB2–SOS signaling, and (ii) markers of pre-existing or readily inducible bypass circuits. Current evidence supports the following practical hypotheses (Riedl et al., 2025; Thatikonda et al., 2024):

- Higher likelihood of benefit: RTK-high states (strong phospho-RTK and GRB2 signaling), contexts with prominent ERK rebound signatures under MAPK-targeted therapy, and settings where preclinical co-targeting data show clear vertical synergy (e.g., KRASG12C plus SOS1; EGFR-mutant spheroid models) (Amodio et al., 2020; Thatikonda et al., 2024; Theard et al., 2020).
- Higher likelihood of rapid escape: pre-existing downstream MAPK alterations (RAF/MEK mutations or fusions), polyclonal RAS/MAPK alterations emerging under pressure (multiple concurrent clones), and strong PI3K/AKT or adhesion-driven survival programs that can sustain viability when RAS-GTP is partially lowered (Mohanty et al., 2023; Riedl et al., 2025; Yaeger et al., 2023).
- Potential modifiers: SOS2 dependence or high SOS2 activity (tissue- and genotype-dependent) may blunt SOS1 selectivity, while EMT and plasticity programs can re-route signaling through alternative RTKs (e.g., FGFR) and reduce the depth or durability of MAPK suppression (Kitai et al., 2016; Sheffels et al., 2018; Theard et al., 2020; Zak et al., 2025).

## 11 Clinical development status and trial design considerations

The central translational promise of KRAS–SOS1 protein–protein interaction (PPI) disruption is that it targets the nucleotide-loading step that feeds all KRAS alleles and can be paired with allele-specific or downstream inhibitors to deepen pathway suppression. In practice, however, SOS1 inhibition is being tested clinically in a landscape where KRAS-mutant tumors are already treated with KRAS (G12C) inhibitors (where available) and where adaptive feedback through RTKs can rapidly restore RAS-GTP and ERK signaling. Early-phase clinical programs therefore emphasize (i) direct evidence of pathway modulation in patients, and (ii) rational combination designs that counteract rebound signaling (for example, with MEK inhibitors) or prevent KRAS (G12C) “reloading” after covalent inhibition.

### 11.1 SOS1 inhibitors in the clinic: current landscape

**FIGURE 9:**
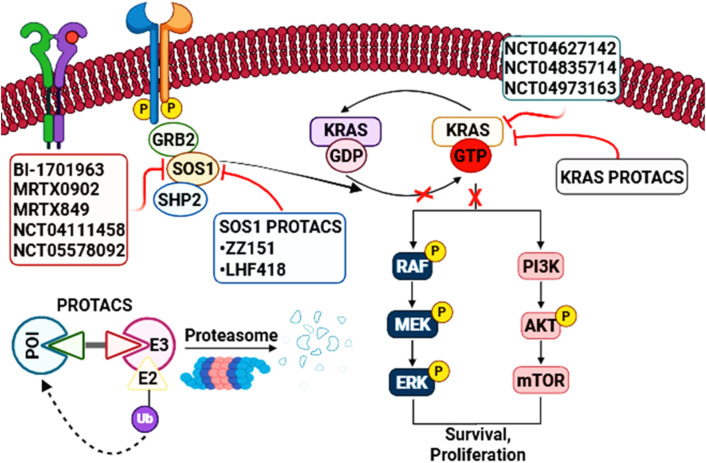
KRAS signaling pathway and representative targeted agents in clinical development. Schematic depiction of RTK-driven activation of the GRB2–SOS1–SHP2 module and GDP–GTP cycling of KRAS, leading to downstream signaling through the RAF–MEK–ERK and PI3K–AKT–mTOR pathways that support tumor cell survival and proliferation. Boxes highlight examples of clinical-stage inhibitors targeting proximal regulators (SOS1/SHP2) and KRAS, along with emerging PROTAC-based degraders directed against SOS1 or KRAS; selected clinical trial identifiers (NCT numbers) are shown where available. Red inhibitory symbols indicate points of pharmacologic intervention.

As of early 2026, clinical testing has focused on two SOS1-directed small-molecule programs: the Boehringer Ingelheim SOS1 inhibitor BI-1701963 and the Mirati/Bristol Myers Squibb SOS1 inhibitor MRTX0902. BI-1701963 is being evaluated in a first-in-human dose-escalation trial as monotherapy and in combination with trametinib (NCT04111458) (Gort et al., 2020). In parallel, MRTX0902 entered a Phase 1/2 program that includes monotherapy in tumors with KRAS/MAPK-pathway alterations and combination therapy with adagrasib (MRTX849) in KRAS (G12C)-mutant disease (NCT05578092) (Boilève et al., 2024; Sudhakar et al., 2024) (Figure 9).

### 11.2 BI-1701963: first-in-human program and early clinical signals

BI-1701963 was designed as a pan-KRAS pathway modulator by binding a pocket on SOS1 that is functionally coupled to the KRAS–SOS1 interface, thereby reducing productive SOS1-mediated nucleotide exchange and lowering RAS-GTP formation (Gort et al., 2020). Because SOS1 blockade is expected to be largely cytostatic as a single agent in many settings, the clinical development plan for BI-1701963 has prominently featured combination cohorts intended to suppress feedback-driven rebound signatures or to reinforce KRAS GDP-state occupancy (for KRAS (G12C) inhibitors) (Gort et al., 2020) (Figure 9).

In addition to the trametinib combination arm within NCT04111458, related phase I studies were registered to evaluate BI-1701963 with irinotecan in KRAS-mutant colorectal cancer (NCT04627142) (Gerlach et al., 2020), with the MEK inhibitor BI 3011441 (NCT04835714), and with the irreversible KRAS (G12C) inhibitor BI 1823911 (NCT04973163) (Heymach et al., 2023). These designs reflect a consistent hypothesis: upstream blockade of nucleotide exchange can (i) blunt RTK-driven reactivation of RAS signaling after pathway inhibition and (ii) increase the fraction of KRAS (G12C) in the GDP state that is accessible to covalent inhibitors (Figure 9).

Publicly available efficacy reporting for BI-1701963 remains limited. A snapshot analysis of the BI-1701963 dose-escalation experience in KRAS-mutant solid tumors has been presented at ESMO 2021 (abstract 524P) (Johnson et al., 2021), and additional “trial in process” updates describing combination cohorts have been disseminated through meeting abstracts (Hofmann et al., 2021). At this stage, the most consistent takeaway is that clinical development is being positioned around combination benefit and on-target pathway modulation rather than expectations of high single-agent response rates.

### 11.3 MRTX0902: next-generation SOS1 inhibitor entering phase 1/2

MRTX0902 represents a medicinal-chemistry advance toward clinical-candidate-quality SOS1 inhibition (potency, selectivity, and oral exposure) and is explicitly framed as “combination-ready” based on preclinical pharmacology (Ketcham et al., 2022). In the ongoing Phase 1/2 trial (NCT05578092), the monotherapy portion enrolls patients with solid tumors harboring KRAS mutations or other oncogenic alterations in the KRAS/MAPK pathway (including activating SOS1/PTPN11/class III BRAF/EGFR alterations or inactivating NF1), while combination therapy cohorts evaluate MRTX0902 with adagrasib in KRAS (G12C)-mutant disease (Dillon et al., 2021; Sudhakar et al., 2024) (Figure 9).

A notable design feature of NCT05578092 is the requirement for baseline and on-treatment tumor biopsies for pharmacodynamic evaluation when medically feasible, supporting a rigorous linkage between drug exposure and pathway modulation in humans (Dillon et al., 2021; Sudhakar et al., 2024). This biopsy-driven approach aligns with the mechanism-of-action claim for SOS1 inhibitors (lowering RAS-GTP) and provides a framework for validating whether biochemical engagement translates into durable suppression of downstream outputs such as pERK in different tumor contexts.

### 11.4 Trial design considerations for SOS1 inhibition and combinations

#### Dose escalation and combination lead-in

For both BI-1701963- and MRTX0902-based regimens, early-phase trials prioritize determination of a recommended phase 2 dose (RP2D) using standard safety, dose-limiting toxicity, and pharmacokinetic endpoints (Dillon et al., 2021; Gort et al., 2020; Sudhakar et al., 2024). Combination cohorts typically require a staged approach (monotherapy dose finding followed by combination escalation or safety lead-in) to manage overlapping toxicities and to disentangle exposure contributions from each agent - especially when pairing SOS1 inhibitors with MEK inhibitors, which have well-described dermatologic and gastrointestinal adverse-event profiles in KRAS-mutant populations (Blumenschein et al., 2015).

#### Pharmacodynamic strategy: proving target modulation in patients

Given that SOS1 inhibitors are intended to reduce RAS-GTP and thereby suppress pathway output, pharmacodynamic (PD) endpoints are central to “go/no-go” decision making. Across the literature, the most directly mechanistic PD readouts are: (i) reduction in RAS-GTP in tumor lysates or surrogate tissues, (ii) downstream signaling suppression (for example, pERK and pathway-adjacent markers such as pS6), and (iii) molecular response monitoring by circulating tumor DNA (circulating tumor DNA (ctDNA)). The mandatory paired-biopsy design in NCT05578092 is therefore strategically important for validating on-target pathway modulation in humans rather than inferring it solely from preclinical models (Dillon et al., 2021; Sudhakar et al., 2024).

#### Patient selection and hypothesis-driven expansion cohorts

SOS1 inhibitors sit upstream of KRAS and can, in principle, affect both mutant and wild-type RAS signaling; however, clinical benefit is expected to be context dependent. Two pragmatic enrichment axes are emerging from trial eligibility and the broader biology: (i) genotype-defined dependence (KRAS-mutant tumors and selected KRAS/MAPK pathway alterations), and (ii) signaling tone at baseline, including RTK activity that can drive high nucleotide exchange and rapid rebound after pathway blockade (Dillon et al., 2021; Sudhakar et al., 2024). Accordingly, expansion cohorts are commonly organized by tumor type and KRAS allele (or KRAS (G12C) for combination with adagrasib), with the expectation that SOS1 inhibition will be most impactful where upstream flux meaningfully contributes to maintaining RAS-GTP.

### 11.5 Practical endpoints and tolerability considerations

From a clinical operations standpoint, SOS1 inhibitor trials must balance mechanistic PD depth with feasibility. Practical considerations include timing of on-treatment biopsies (to capture early RAS-GTP and pERK changes before adaptive rewiring), integration of ctDNA as a minimally invasive response/escape monitor, and dose-scheduling choices for combinations (continuous vs. intermittent) that preserve PD suppression while limiting chronic toxicities (Blumenschein et al., 2015; Dillon et al., 2021; Sudhakar et al., 2024). Importantly, because the therapeutic thesis for SOS1 inhibitors is largely “multiplier” behavior in combination settings, trial designs that explicitly connect PD modulation to combination efficacy - rather than reporting response rates in isolation - will be most informative for defining where SOS1 blockade adds durable clinical value.

An important translational issue is whether SOS1 inhibition can achieve a sufficient therapeutic window in patients, particularly in combination regimens. From an on-target perspective, SOS1/SOS2 biology suggests both opportunity and caution: the relative dispensability of SOS2 and the frequent dominance of SOS1 in sustaining KRAS-driven signaling support the rationale for selective SOS1 blockade, which may preserve some physiologic Ras-exchange capacity compared with broader SOS-family suppression. However, partial redundancy also implies that the efficacy and tolerability consequences of SOS1 inhibition are likely to be context dependent across tissues and signaling states. In parallel, several SOS1 inhibitor chemotypes emerged from kinase-like heteroaromatic scaffolds, making kinase off-target selectivity a clinically relevant safety consideration rather than a purely medicinal-chemistry concern. Accordingly, translation of this class will depend not only on achieving deep suppression of RAS-GTP, but also on maintaining clean kinase selectivity and adequate tolerability margins for chronic or combination dosing (Baltanás et al., 2025; Daley et al., 2025; Esteban et al., 2000; He et al., 2023; Ketcham et al., 2022; Sheffels et al., 2018; Sudhakar et al., 2024; Zhang et al., 2022).

## 12 Where the field is heading

Across the last decade, SOS1 PPI disruptors progressed from biochemical probes to orally bioavailable agents that can suppress RAS-GTP and dampen downstream MAPK signaling in KRAS-driven models (Daley et al., 2025; Ketcham et al., 2022; Sudhakar et al., 2024). This maturation has clarified a practical thesis for the next wave of programs: SOS1 inhibition is most valuable when it is engineered for combination depth (sustained intracellular target engagement and coverage through feedback) and deployed in biomarker-aware settings where RAS cycling remains rate-limiting (Daley et al., 2025; Sudhakar et al., 2024; Thatikonda et al., 2024).

### 12.1 Next-generation SOS1 inhibitors

The most straightforward trajectory is incremental but important chemistry: maintaining high-affinity binding in the SOS1 pocket while improving permeability, PK margins, and selectivity profiles that support multi-drug regimens (Daley et al., 2025; Ketcham et al., 2022; Sudhakar et al., 2024). In this context, brain penetration is emerging as an explicit design goal for some series, motivated by the high incidence of central nervous system (CNS) metastases in KRAS-mutant lung cancer and by the broader oncology trend toward CNS-inclusive development. MRTX0902 was reported as a potent, selective, brain-penetrant and orally bioavailable SOS1 binder, establishing feasibility for BBB-competent SOS1-pocket chemistry (Ketcham et al., 2022).

Patent disclosures and commentary also point to continued scaffold exploration aimed at CNS exposure and improved physicochemical balance, including 8-aza quinazoline series described as brain-penetrant SOS1 inhibitors (Sabnis, 2023). Beyond CNS coverage, a recurring medicinal chemistry tension is potency versus permeability: many SOS1 binders rely on polar interaction networks to anchor in the pocket, but cytosolic exposure and sustained occupancy in tumors often demand careful control of lipophilicity, polar surface area, and efflux liability (Ketcham et al., 2022; Xiao et al., 2024).

A parallel optimization theme is functional selectivity: minimizing off-target liabilities while preserving the desired systems-level effect, namely, lowering mutant and/or WT RAS-GTP and attenuating rebound signaling when combined with pathway inhibitors (Daley et al., 2025; Xiao et al., 2024). Because SOS1 and SOS2 can be partially redundant in some contexts, future programs will likely stratify indications by the relative contribution of SOS1-mediated exchange (and compensatory SOS2 activity), rather than assuming uniform pan-RAS vulnerability (Theard et al., 2024; Xiao et al., 2024).

### 12.2 SOS1 degraders and other event-driven strategies

Event-driven approaches offer a conceptually distinct route to durable pathway control: instead of requiring continuous pocket occupancy to block productive KRAS–SOS1 complex formation, a degrader can eliminate SOS1 protein and potentially extend pharmacodynamic effects beyond plasma exposure (Bian et al., 2022; Li H. et al., 2024; Luo et al., 2025; Lv et al., 2024; Zhou et al., 2023). Early work demonstrated that BI-3406-derived PROTACs can drive substantial SOS1 degradation with high specificity and translate into improved antiproliferative activity in KRAS-mutant colorectal cancer cell lines and patient-derived organoids (Bian et al., 2022) (Figure 9).

More recent degraders have strengthened the case that SOS1 removal can be combination-enabling. A J. Med. Chem. Report described a potent SOS1 PROTAC that synergized with a KRAS (G12C) inhibitor, consistent with the idea that eliminating SOS1 reduces the nucleotide exchange capacity that supports KRAS “reloading” under drug pressure (Lv et al., 2024). Related series—including LHF418 and ZZ151—underscore that linker engineering and ternary complex cooperativity can materially alter degradation efficiency and *in vivo* efficacy, with reported activity across multiple KRAS mutant backgrounds (Li H. et al., 2024; Zhou et al., 2023) (Figure 9).

An especially forward-looking dataset is the Cancer Research report of SIAIS562055, a BI-3406-analog-based SOS1 PROTAC that produced sustained SOS1 degradation, stronger ERK pathway suppression than small-molecule inhibition, and tumor regression in KRAS-mutant xenografts when combined with KRAS inhibitors—importantly including settings described as having acquired resistance (Luo et al., 2025). Together, these studies suggest that (i) event-driven SOS1 targeting can deepen MAPK suppression, and (ii) degraders may provide a practical way to separate pharmacodynamic durability from peak exposure, albeit with the usual PROTAC challenges (size, oral bioavailability, tissue distribution, and E3-ligase context) (Bian et al., 2022; Li H. et al., 2024; Luo et al., 2025; Lv et al., 2024; Zhou et al., 2023).

### 12.3 Rational multi-target strategies

The clearest multi-target strategy is pairing SOS1 inhibition with direct KRAS inhibitors to block the “reloading” and rewiring that emerges when KRAS is pharmacologically trapped (Daley et al., 2025; Sudhakar et al., 2024; Thatikonda et al., 2024). In a Nature Cancer study, BI-3406 combined with adagrasib produced stronger suppression of RAS–MAPK signaling and more durable antitumor responses than adagrasib alone, delayed the emergence of acquired resistance, and retained activity in adagrasib-resistant models; mechanistically, the authors implicated MRAS activity and the MRAS partner SHOC2 as contributors to resistance that remained suppressible by SOS1 (or SHP2) inhibition (Thatikonda et al., 2024). The same logic generalizes beyond KRAS (G12C). Preclinical work combining SOS1 inhibitors with KRAS (G12D) inhibitors has shown that upstream exchange blockade can amplify the depth of MAPK suppression and increase durability of response in KRAS (G12D)-driven settings, supporting a broader “KRAS + nucleotide-exchange control” framework as more allele-specific KRAS drugs enter the clinic (Baltanás et al., 2025).

A second axis is proximal pathway co-targeting (SOS1 + RTK or SOS1 + SHP2) to shut down feedback-driven activation of WT RAS and limit pathway rebound (Daley et al., 2025; Thatikonda et al., 2024). The Nature Cancer study noted that KRAS (G12C) inhibitor combinations with SOS1 inhibition were comparable in strength to combinations with SHP2 or EGFR inhibitors in tested models, underscoring that multiple proximal nodes can serve as “exchange throttles” in resistant states (Thatikonda et al., 2024). In practice, choosing among these upstream partners will likely depend on tumor lineage, dominant RTKs, and tolerability when combined with KRAS or MEK inhibition (Daley et al., 2025; Thatikonda et al., 2024).

Finally, event-driven SOS1 degraders may themselves become multi-target enablers: SIAIS562055 potentiated both KRAS inhibitors (in KRAS-mutant tumors) and ABL inhibitors (in BCR–ABL-driven leukemia) by sustained disruption of SOS1-dependent signaling programs.86 While these applications are early, they illustrate how SOS1 control can be layered with orthogonal oncogenic drivers when SOS1 sits at a functional bottleneck (Luo et al., 2025).

### 12.4 Key unanswered questions

Who benefits most from SOS1 blockade? Even within KRAS-mutant cancers, SOS1 dependence varies with allele, tissue context, and the degree to which upstream RTKs actively drive nucleotide exchange (Theard et al., 2024; Xiao et al., 2024). A central translational goal is to define “exchange-addicted” states—tumors where RAS-GTP levels are tightly coupled to SOS1 activity—versus states where alternative inputs (e.g., SOS2 or other GEFs) can sustain signaling (Theard et al., 2024; Xiao et al., 2024).

Which combinations win in patients? Preclinical evidence supports pairing SOS1 inhibition with MEK inhibitors and with KRAS allele-specific inhibitors, but optimal scheduling (continuous versus intermittent SOS1 dosing, staggered vs. concurrent start) and regimen selection will likely hinge on managing MAPK-pathway toxicities while maintaining durable pathway suppression (Daley et al., 2025; Sudhakar et al., 2024).

Can resistance be forecasted and preempted? Mechanistic studies point to upstream rewiring (RTK/SHP2 dependence) and to MRAS–SHOC2 pathway engagement as plausible escape routes under KRAS (G12C) pressure, with SOS1 inhibition retaining activity against some resistant states (Thatikonda et al., 2024). More broadly, resistance may emerge through reactivation of ERK signaling or through pathway switching toward PI3K/AKT, and a practical agenda is to pair early clinical PD (RAS-GTP, pERK dynamics, transcriptional rebound signatures) with longitudinal genomics to identify dominant escape circuits in real time (Daley et al., 2025; Sudhakar et al., 2024; Theard et al., 2024).

## 13 Conclusion

KRAS–SOS1 PPI disruption has matured from a structural hypothesis into a pharmacologically actionable strategy: multiple chemotypes bind an allosteric pocket on SOS1, destabilize productive Ras–SOS engagement, and lower RAS-GTP with downstream pERK suppression in cells and tumors. These agents are rarely maximally cytotoxic alone, but they consistently function as pathway “multipliers” by limiting feedback-driven reactivation and by increasing the GDP-state fraction that benefits KRAS allele-specific inhibitors. The strongest preclinical evidence supports vertical combinations—especially with MEK inhibitors and KRAS (G12C) or KRAS (G12D) inhibitors—where upstream exchange control deepens and extends MAPK pathway blockade and can delay acquired resistance. Event-driven strategies (SOS1 degraders) now add a second modality layer, showing that sustained SOS1 removal can enhance ERK suppression and sensitize resistant KRAS-mutant tumors to KRAS inhibitors.82-86 Clinically, early SOS1 programs are testing whether these mechanistic advantages translate into tolerable, biomarker-guided regimens in patients. Looking ahead, key priorities are to (i) identify exchange-dependent tumor states and robust PD markers, (ii) determine which proximal partners (RTK, SHP2, SOS1) most safely and effectively deepen KRAS inhibition in each lineage, and (iii) anticipate resistance through integrated pharmacodynamic and genomic monitoring. If these questions are answered, SOS1 inhibition could become a broadly deployable component of combination therapy for KRAS-driven cancers rather than a niche add-on for any single allele.

## References

[B1] Ahmed T. A. Adamopoulos C. Karoulia Z. Wu X. Sachidanandam R. Aaronson S. A. (2019). SHP2 drives adaptive resistance to ERK signaling inhibition in molecularly defined subsets of ERK-dependent tumors. Cell Rep. 26, 65–78.e65. 10.1016/j.celrep.2018.12.013 30605687 PMC6396678

[B2] Amodio V. Yaeger R. Arcella P. Cancelliere C. Lamba S. Lorenzato A. (2020). EGFR blockade reverts resistance to KRASG12C inhibition in colorectal cancer. Cancer Discov. 10, 1129–1139. 10.1158/2159-8290.CD-20-0187 32430388 PMC7416460

[B3] Baines A. T. Xu D. Der C. J. (2011). Inhibition of ras for cancer treatment: the search continues. Future Med. Chem. 3, 1787–1808. 10.4155/fmc.11.121 22004085 PMC3347641

[B4] Baltanás F. C. Kramer-Drauberg M. García-Navas R. Patrucco E. Petrini E. Arnhof H. (2025). SOS1 inhibitor BI-3406 shows *in vivo* antitumor activity akin to genetic ablation and synergizes with a KRAS^G12D^ inhibitor in KRAS LUAD. Proc. Natl. Acad. Sci. 122 **,** e2422943122, 10.1073/pnas.2422943122 40073053 PMC11929440

[B5] Bandaru P. Kondo Y. Kuriyan J. (2019). The interdependent activation of son-of-sevenless and ras. Cold Spring Harb. Perspect. Med. 9, a031534. 10.1101/cshperspect.a031534 29610148 PMC6360870

[B6] Bian Y. Alem D. Beato F. Hogenson T. L. Yang X. Jiang K. (2022). Development of SOS1 inhibitor-based degraders to target KRAS-mutant colorectal cancer. J. Med. Chem. 65, 16432–16450. 10.1021/acs.jmedchem.2c01300 36459180 PMC10113742

[B8] Blumenschein G. R. Smit E. F. Planchard D. Kim D. W. Cadranel J. De Pas T. (2015). A randomized phase II study of the MEK1/MEK2 inhibitor trametinib (GSK1120212) compared with docetaxel in KRAS-mutant advanced non-small-cell lung cancer (NSCLC). Ann. Oncol. 26, 894–901. 10.1093/annonc/mdv072 25722381 PMC4855243

[B9] Boilève A. Smolenschi C. Lambert A. Boige V. Delaye M. Camilleri G. M. (2024). KRAS, a new target for precision medicine in colorectal cancer? Cancers 16, 3455. 10.3390/cancers16203455 39456549 PMC11506008

[B10] Boriack-Sjodin P. A. Margarit S. M. Bar-Sagi D. Kuriyan J. (1998). The structural basis of the activation of Ras by Sos. Nature 394, 337–343. 10.1038/28548 9690470

[B11] Burns M. C. Sun Q. Daniels R. N. Camper D. Kennedy J. P. Phan J. (2014). Approach for targeting Ras with small molecules that activate SOS-mediated nucleotide exchange. Proc. Natl. Acad. Sci. U. S. A. 111. 3401–3406. 10.1073/pnas.1315798111 24550516 PMC3948241

[B12] Chardin P. Camonis J. H. Gale N. W. Van Aelst L. Schlessinger J. Wigler M. H. (1993). Human Sos1: a guanine nucleotide exchange factor for ras that binds to GRB2. Science 260, 1338–1343. 10.1126/science.8493579 8493579

[B13] Christensen S. M. Tu H.-L. Jun J. E. Alvarez S. Triplet M. G. Iwig J. S. (2016). One-way membrane trafficking of SOS in receptor-triggered Ras activation. Nat. Struct. and Mol. Biol. 23, 838–846. 10.1038/nsmb.3275 27501536 PMC5016256

[B14] Cooper G. M. (1982). Cellular transforming genes. Science 217, 801–806. 10.1126/science.6285471 6285471

[B15] Corbalan-Garcia S. Yang S. S. Degenhardt K. R. Bar-Sagi D. (1996). Identification of the mitogen-activated protein kinase phosphorylation sites on human sosl that regulate interaction with Grb2. Mol. Cell. Biol. 16, 5674–5682. 10.1128/mcb.16.10.5674 8816480 PMC231567

[B16] Cox A. D. Fesik S. W. Kimmelman A. C. Luo J. Der C. J. (2014). Drugging the undruggable RAS: mission Possible? Nat. Rev. Drug Discov. 13, 828–851. 10.1038/nrd4389 25323927 PMC4355017

[B17] Daley B. R. Vieira H. M. Rao C. Hughes J. M. Beckley Z. M. Huisman D. H. (2023). SOS1 and KSR1 modulate MEK inhibitor responsiveness to target resistant cell populations based on PI3K and KRAS mutation status, Proc. Natl. Acad. Sci. U. S. A. 120 **,** e2313137120, 10.1073/pnas.2313137120 37972068 PMC10666034

[B18] Daley B. R. Sealover N. E. Finniff B. A. Hughes J. M. Sheffels E. Gerlach D. (2025). SOS1 inhibition enhances the efficacy of KRASG12C inhibitors and delays resistance in lung adenocarcinoma. Cancer Res. 85, 118–133. 10.1158/0008-5472.CAN-23-3256 39437166 PMC11695159

[B19] Dillon M. Lopez A. Lin E. Sales D. Perets R. Jain P. (2021). Progress on Ras/MAPK signaling research and targeting in blood and solid cancers. Cancers 13, 5059. 10.3390/cancers13205059 34680208 PMC8534156

[B20] Esteban L. M. Fernández-Medarde A. López E. Yienger K. Guerrero C. Ward J. M. (2000). Ras-Guanine nucleotide exchange factor Sos2 is dispensable for mouse growth and development. Mol. Cell. Biol. 20, 6410–6413. 10.1128/mcb.20.17.6410-6413.2000 10938118 PMC86116

[B21] Evelyn C. r. Duan X. Biesiada J. Seibel W. l. Meller J. Zheng Y. (2014). Rational design of small molecule inhibitors targeting the ras GEF, SOS1. Chem. and Biol. 21, 1618–1628. 10.1016/j.chembiol.2014.09.018 25455859 PMC4272618

[B22] Evelyn C. R. Biesiada J. Duan X. Tang H. Shang X. Papoian R. (2015). Combined rational design and a high throughput screening platform for identifying chemical inhibitors of a ras-activating enzyme. J. Biol. Chem. 290, 12879–12898. 10.1074/jbc.M114.634493 25825487 PMC4432303

[B23] Fedele C. Li S. Teng K. W. Foster C. J. R. Peng D. Ran H. (2021). SHP2 inhibition diminishes KRASG12C cycling and promotes tumor microenvironment remodeling. J. Exp. Med. 218 (1), e20201414. 10.1084/jem.20201414 33045063 PMC7549316

[B24] Freedman T. S. Sondermann H. Friedland G. D. Kortemme T. Bar-Sagi D. Marqusee S. (2006). A ras-induced conformational switch in the ras activator Son of sevenless. Proc. Natl. Acad. Sci. 103, 16692–16697. 10.1073/pnas.0608127103 17075039 PMC1629002

[B25] Gerlach D. Gmachl M. Ramharter J. Teh J. Fu S.-C. Trapani F. (2020). Abstract 1091: BI-3406 and BI 1701963: potent and selective SOS1::KRAS inhibitors induce regressions in combination with MEK inhibitors or irinotecan. Cancer Res. 80, 1091. 10.1158/1538-7445.am2020-1091

[B26] Gort E. Johnson M. L. Hwang J. J. Pant S. Dünzinger U. Riemann K. (2020). A phase I, open-label, dose-escalation trial of BI 1701963 as monotherapy and in combination with trametinib in patients with KRAS mutated advanced or metastatic solid tumors. J. Clin. Oncol. 38, TPS3651. 10.1200/jco.2020.38.15_suppl.tps3651

[B27] Gureasko J. Kuchment O. Makino D. L. Sondermann H. Bar-Sagi D. Kuriyan J. (2010). Role of the histone domain in the autoinhibition and activation of the Ras activator Son of sevenless. Proc. Natl. Acad. Sci. 107, 3430–3435. 10.1073/pnas.0913915107 20133692 PMC2816639

[B28] Guthof N. Xue H. Jerchel-Furau I. Hethey C. Busse J.-E. Denner K. (2025). Abstract 4373: discovery and characterization of BAY 3498264: a small molecule inhibitor targeting the RAS-SOS1 interaction. Cancer Res. 85, 4373. 10.1158/1538-7445.am2025-4373

[B29] Haddad S. F. Bouferraa Y. Nair K. G. (2025). Adagrasib in the treatment of colorectal cancer. Future Oncol. 21, 2275–2285. 10.1080/14796694.2025.2524311 40619745 PMC12323406

[B30] Hallin J. Bowcut V. Calinisan A. Briere D. M. Hargis L. Engstrom L. D. (2022). Anti-tumor efficacy of a potent and selective non-covalent KRASG12D inhibitor. Nat. Med. 28, 2171–2182. 10.1038/s41591-022-02007-7 36216931

[B31] He H. Zhang Y. Xu J. Li Y. Fang H. Liu Y. (2022). Discovery of orally bioavailable SOS1 inhibitors for suppressing KRAS-Driven carcinoma. J. Med. Chem. 65, 13158–13171. 10.1021/acs.jmedchem.2c00986 36173339

[B32] He H. Chen R. Wang Z. Qing L. Zhang Y. Liu Y. (2023). Design of orally-bioavailable Tetra-cyclic phthalazine SOS1 inhibitors with high selectivity against EGFR. Bioorg. Chem. 136, 106536. 10.1016/j.bioorg.2023.106536 37054529

[B33] Heymach J. Kotecki N. Prenen H. Alonso G. Lindsay C. R. Barve M. (2023). 665P first-in-human, phase Ia/b, dose-escalation/expansion study of KRAS G12C inhibitor BI 1823911, as monotherapy and combined with anticancer therapies, in patients (pts) with advanced or metastatic solid tumours harbouring a KRAS G12C mutation. Ann. Oncol. 34, S468. 10.1016/j.annonc.2023.09.1851

[B34] Hillig R. C. Sautier B. Schroeder J. Moosmayer D. Hilpmann A. Stegmann C. M. (2019). Discovery of potent SOS1 inhibitors that block RAS activation *via* disruption of the RAS-SOS1 interaction, Proc. Natl. Acad. Sci. U. S. A. 116 **,** 2551–2560. 10.1073/pnas.1812963116 30683722 PMC6377443

[B35] Hobbs G. A. Der C. J. Rossman K. L. (2016). RAS isoforms and mutations in cancer at a glance. J. Cell Sci. 129, 1287–1292. 10.1242/jcs.182873 26985062 PMC4869631

[B36] Hofmann M. H. Gmachl M. Ramharter J. Savarese F. Gerlach D. Marszalek J. R. (2021). BI-3406, a potent and selective SOS1–KRAS interaction inhibitor, is effective in KRAS-Driven cancers through combined MEK inhibition. Cancer Discov. 11, 142–157. 10.1158/2159-8290.CD-20-0142 32816843 PMC7892644

[B37] Isermann T. Sers C. Der C. J. Papke B. (2025). KRAS inhibitors: resistance drivers and combinatorial strategies. Trends Cancer 11, 91–116. 10.1016/j.trecan.2024.11.009 39732595 PMC13308734

[B38] Jänne P. A. Riely G. J. Gadgeel S. M. Heist R. S. Ou S.-H. I. Pacheco J. M. (2022). Adagrasib in Non–Small-Cell lung cancer harboring a KRASG12C mutation. N. Engl. J. Med. 387, 120–131. 10.1056/NEJMoa2204619 35658005

[B39] Johnson M. L. Gort E. Pant S. Lolkema M. P. Sebastian M. Scheffler M. (2021). 524P A phase I, open-label, dose-escalation trial of BI 1701963 in patients (pts) with KRAS mutated solid tumours: a snapshot analysis. Ann. Oncol. 32, S591–S592. 10.1016/j.annonc.2021.08.1046

[B40] Karnoub A. E. Weinberg R. A. (2008). Ras oncogenes: split personalities. Nat. Rev. Mol. Cell Biol. 9, 517–531. 10.1038/nrm2438 18568040 PMC3915522

[B41] Ketcham J. M. Haling J. Khare S. Bowcut V. Briere D. M. Burns A. C. (2022). Design and discovery of MRTX0902, a potent, selective, Brain-Penetrant, and orally bioavailable inhibitor of the SOS1:KRAS protein-protein interaction. J. Med. Chem. 65, 9678–9690. 10.1021/acs.jmedchem.2c00741 35833726 PMC9340770

[B42] Kitai H. Ebi H. Tomida S. Floros K. V. Kotani H. Adachi Y. (2016). Epithelial-to-Mesenchymal transition defines feedback activation of receptor tyrosine kinase signaling induced by MEK inhibition in KRAS-Mutant lung cancer. Cancer Discov. 6, 754–769. 10.1158/2159-8290.CD-15-1377 27154822 PMC4957999

[B43] Knickelbein K. Zhang L. (2015). Mutant KRAS as a critical determinant of the therapeutic response of colorectal cancer. Genes and Dis. 2, 4–12. 10.1016/j.gendis.2014.10.002 25815366 PMC4372129

[B44] Koga T. Suda K. Fujino T. Ohara S. Hamada A. Nishino M. (2021). KRAS secondary mutations that confer acquired resistance to KRAS G12C inhibitors, Sotorasib and Adagrasib, and overcoming strategies: insights from *in vitro* experiments. J. Thorac. Oncol. 16, 1321–1332. 10.1016/j.jtho.2021.04.015 33971321

[B45] Kolch W. Berta D. Rosta E. (2023). Dynamic regulation of RAS and RAS signaling. Biochem. J. 480, 1–23. 10.1042/BCJ20220234 36607281 PMC9988006

[B46] Lambert Q. T. Reuther G. W. (2006). “Activation of ras proteins by ras Guanine Nucleotide releasing protein family members,” in Methods in enzymology (Academic Press), 82–98. 10.1016/S0076-6879(05)07008-4 16757316

[B47] Leffler A. E. Houang E. M. Gray F. Placzek A. T. Ruvinsky A. M. Bell J. A. (2025). Exploiting solvent exposed salt-bridge interactions for the discovery of potent inhibitors of SOS1 using free-energy perturbation simulations. ACS Med. Chem. Lett. 16, 444–453. 10.1021/acsmedchemlett.4c00602 40104782 PMC11912288

[B48] Leshchiner E. S. Parkhitko A. Bird G. H. Luccarelli J. Bellairs J. A. Escudero S. (2015). Direct inhibition of oncogenic KRAS by hydrocarbon-stapled SOS1 helices. Proc. Natl. Acad. Sci. U. S. A. 112, 1761–1766. 10.1073/pnas.1413185112 25624485 PMC4330742

[B49] Li A. Li X. Zou J. Zhuo X. Chen S. Chai X. (2023). SOS1-inspired hydrocarbon-stapled peptide as a pan-Ras inhibitor. Bioorg. Chem. 135, 106500. 10.1016/j.bioorg.2023.106500 37003134

[B50] Li D. Xie Q. Yang M. Cai Y. Sun K. Jiang S. (2024). Lead identification of novel naphthyridine derivatives as potent SOS1 inhibitors. ACS Med. Chem. Lett. 15, 958–964. 10.1021/acsmedchemlett.4c00156 38894918 PMC11181497

[B51] Li H. Chai M. Chen Y. Zhou F. Ren X. Xu J. (2024). Discovery of LHF418 as a new potent SOS1 PROTAC degrader. Bioorg. and Med. Chem. 103, 117661. 10.1016/j.bmc.2024.117661 38489998

[B52] Li D. Xie Q. Yang M. Sun K. Jiang S. Yu S. (2025). Identification of novel 2-(Azetidin-3-ylamino)nicotinamide derivatives as potent KRAS::SOS1 inhibitors. ACS Med. Chem. Lett. 16, 1626–1633. 10.1021/acsmedchemlett.5c00300 40832539 PMC12359004

[B53] Li J. Song N. Ma R. Qu X. (2026). Unravelling resistance: integrating metabolism, epigenetics, immunology, and proteostasis in strategies against Kirsten Rat Sarcoma Viral Oncogene Homolog-mutant colorectal cancer. Int. J. Biol. Sci. 22, 426–446. 10.7150/ijbs.118831 41362725 PMC12681943

[B54] Liu M. Zhou G. Su W. Gu Y. Gao M. Wang K. (2023). Design, synthesis, and bioevaluation of Pyrido[2,3-d]pyrimidin-7-ones as potent SOS1 inhibitors. ACS Med. Chem. Lett. 14, 183–190. 10.1021/acsmedchemlett.2c00490 36793426 PMC9923844

[B55] Luo Z. Lin C. Yu C. Yuan C. Wu W. Xu X. (2025). Targeted degradation of SOS1 exhibits potent anticancer activity and overcomes resistance in KRAS-Mutant tumors and BCR–ABL–Positive leukemia. Cancer Res. 85, 101–117. 10.1158/0008-5472.CAN-24-1093 39437162 PMC11694061

[B56] Lv Y. Yang Z. Chen Y. Ma X. Guo M. Zhang C. (2024). A potent SOS1 PROTAC degrader with synergistic efficacy in combination with KRASG12C inhibitor. J. Med. Chem. 67, 2487–2511. 10.1021/acs.jmedchem.3c01598 38316747

[B57] Malhotra J. Li X. Palmer J. Synold T. W. Mohanty A. Singhal S. (2024). EP.12H.07 A phase I clinical trial of Carfilzomib in combination with Sotorasib in patients with KRASG12C mutated NSCLC. J. Thorac. Oncol. 19, S659–S660. 10.1016/j.jtho.2024.09.1390

[B58] Margarit S. M. Sondermann H. Hall B. E. Nagar B. Hoelz A. Pirruccello M. (2003). Structural evidence for feedback activation by Ras·GTP of the ras-specific nucleotide exchange factor SOS. Cell 112, 685–695. 10.1016/s0092-8674(03)00149-1 12628188

[B59] Mausey N. Halford Z. (2024). Targeted therapies for previously “Undruggable” KRAS-mutated non–small cell lung cancer: a review of sotorasib and Adagrasib. Ann. Pharmacother. 58, 622–635. 10.1177/10600280231197459 37700573

[B60] Mohanty A. Nam A. Srivastava S. Jones J. Lomenick B. Singhal S. S. (2023). Acquired resistance to KRAS G12C small-molecule inhibitors *via* genetic/nongenetic mechanisms in lung cancer. Sci. Adv. 9, eade3816. 10.1126/sciadv.ade3816 37831779 PMC10575592

[B61] Mozzarelli A. M. Simanshu D. K. Castel P. (2024). Functional and structural insights into RAS effector proteins. Mol. Cell 84, 2807–2821. 10.1016/j.molcel.2024.07.024 39025071 PMC11316660

[B62] Ostrem J. M. L. Shokat K. M. (2016). Direct small-molecule inhibitors of KRAS: from structural insights to mechanism-based design. Nat. Rev. Drug Discov. 15, 771–785. 10.1038/nrd.2016.139 27469033

[B63] Parada L. F. Tabin C. J. Shih C. Weinberg R. A. (1982). Human EJ bladder carcinoma oncogene is homologue of Harvey sarcoma virus ras gene. Nature 297, 474–478. 10.1038/297474a0 6283357

[B64] Parums D. V. (2022). Editorial: recent approval of Sotorasib as the first targeted therapy for KRAS G12C-Mutated advanced non-small cell lung cancer (NSCLC). Med. Sci. Monit. 28, e938746. 10.12659/MSM.938746 36317327 PMC9636839

[B65] Prior I. A. Lewis P. D. Mattos C. (2012). A Comprehensive Survey of ras mutations in cancer. Cancer Res. 72, 2457–2467. 10.1158/0008-5472.CAN-11-2612 22589270 PMC3354961

[B66] Prior I. A. Hood F. E. Hartley J. L. (2020). The frequency of ras mutations in cancer. Cancer Res. 80, 2969–2974. 10.1158/0008-5472.CAN-19-3682 32209560 PMC7367715

[B67] Pylayeva-Gupta Y. Grabocka E. Bar-Sagi D. (2011). RAS oncogenes: weaving a tumorigenic web. Nat. Rev. Cancer 11, 761–774. 10.1038/nrc3106 21993244 PMC3632399

[B68] Qian X. Vass W. C. Papageorge A. G. Anborgh P. H. Lowy D. R. (1998). N terminus of Sos1 ras exchange factor: critical roles for the dbl and Pleckstrin homology domains. Mol. Cell. Biol. 18, 771–778. 10.1128/mcb.18.2.771 9447973 PMC108788

[B69] Ramharter J. Kessler D. Ettmayer P. Hofmann M. H. Gerstberger T. Gmachl M. (2021). One atom makes all the difference: getting a foot in the door between SOS1 and KRAS. J. Med. Chem. 64, 6569–6580. 10.1021/acs.jmedchem.0c01949 33719426

[B70] Riedl J. M. Fece De La Cruz F. Lin J. J. Parseghian C. Kim J. E. Matsubara H. (2025). Genomic landscape of clinically acquired resistance alterations in patients treated with KRASG12C inhibitors. Ann. Oncol. 36, 682–692. 10.1016/j.annonc.2025.01.020 39914665 PMC12097956

[B71] Rodenhuis S. (1992). Ras and human tumors. Semin. Cancer Biol. 3, 241–247. 1421168

[B72] Rojas J. M. Santos E. (2006). “Ras-Gefs and ras gaps,” in RAS family GTPases, (Dordrecht: Springer Netherlands), 15–43.

[B73] Ryan M. B. Coker O. Sorokin A. Fella K. Barnes H. Wong E. (2022). KRAS^G12C^-independent feedback activation of wild-type RAS constrains KRAS^G12C^ inhibitor efficacy. Cell Rep. 39, 110993. 10.1016/j.celrep.2022.110993 35732135 PMC9809542

[B74] Sabnis R. W. (2023). 8-Aza quinazolines as brain penetrant SOS1 inhibitors for treating cancer. ACS Med. Chem. Lett. 14, 1487–1488. 10.1021/acsmedchemlett.3c00416 37974940 PMC10641917

[B75] Santos E. Tronick S. R. Aaronson S. A. Pulciani S. Barbacid M. (1982). T24 human bladder carcinoma oncogene is an activated form of the normal human homologue of BALB- and Harvey-MSV transforming genes. Nature 298, 343–347. 10.1038/298343a0 6283384

[B76] Sheffels E. Kortum R. L. (2021). Breaking oncogene addiction: getting RTK/RAS-Mutated cancers off the SOS. J. Med. Chem. 64, 6566–6568. 10.1021/acs.jmedchem.1c00698 33961431

[B77] Sheffels E. Sealover N. E. Wang C. Kim D. H. Vazirani I. A. Lee E. (2018). Oncogenic RAS isoforms show a hierarchical requirement for the guanine nucleotide exchange factor SOS2 to mediate cell transformation. Sci. Signal. 11, eaar8371. 10.1126/scisignal.aar8371 30181243 PMC7396125

[B78] Simanshu D. K. Nissley D. V. Mccormick F. (2017). RAS proteins and their regulators in human disease. Cell 170, 17–33. 10.1016/j.cell.2017.06.009 28666118 PMC5555610

[B79] Skoulidis F. Li B. T. Dy G. K. Price T. J. Falchook G. S. Wolf J. (2021). Sotorasib for lung cancers with *KRAS* p.G12C mutation. N. Engl. J. Med. 384, 2371–2381. 10.1056/NEJMoa2103695 34096690 PMC9116274

[B80] Solanki H. S. Shah H. Imbody D. Desai B. Kato R. Miroshnychenko D. (2026). RAS-GTP inhibition overcomes acquired resistance to KRASG12C inhibitors mediated by oncogenic and wild-type RAS activation in non–small cell lung cancer. Cancer Res. 86, 467–484. 10.1158/0008-5472.CAN-25-0600 41165456 PMC12962340

[B81] Sondermann H. Soisson S. M. Boykevisch S. Yang S.-S. Bar-Sagi D. Kuriyan J. (2004). Structural analysis of autoinhibition in the ras activator son of sevenless. Cell 119, 393–405. 10.1016/j.cell.2004.10.005 15507210

[B82] Sudhakar N. Yan L. Qiryaqos F. Engstrom L. D. Laguer J. Calinisan A. (2024). The SOS1 inhibitor MRTX0902 blocks KRAS activation and demonstrates antitumor activity in cancers dependent on KRAS nucleotide loading. Mol. Cancer Ther. 23, 1418–1430. 10.1158/1535-7163.MCT-23-0870 38904222 PMC11443210

[B83] Sütcüoğlu O. Yıldırım H. Ç. Almuradova E. Günenç D. Yalçın Ş. (2025). RAS mutations in advanced colorectal cancer: mechanisms, clinical implications, and novel therapeutic approaches. Medicina 61, 1202. 10.3390/medicina61071202 40731832 PMC12299888

[B84] Tanaka N. Lin J. J. Li C. Ryan M. B. Zhang J. Kiedrowski L. A. (2021). Clinical acquired resistance to KRASG12C inhibition through a novel KRAS Switch-II pocket mutation and polyclonal alterations converging on RAS–MAPK reactivation. Cancer Discov. 11, 1913–1922. 10.1158/2159-8290.CD-21-0365 33824136 PMC8338755

[B85] Taparowsky E. Suard Y. Fasano O. Shimizu K. Goldfarb M. Wigler M. (1982). Activation of the T24 bladder carcinoma transforming gene is linked to a single amino acid change. Nature 300, 762–765. 10.1038/300762a0 7177195

[B86] Thatikonda V. Lyu H. Jurado S. Kostyrko K. Bristow C. A. Albrecht C. (2024). Co-targeting SOS1 enhances the antitumor effects of KRASG12C inhibitors by addressing intrinsic and acquired resistance. Nat. Cancer 5, 1352–1370. 10.1038/s43018-024-00800-6 39103541 PMC11424490

[B87] Theard P. L. Sheffels E. Sealover N. E. Linke A. J. Pratico D. J. Kortum R. L. (2020). Marked synergy by vertical inhibition of EGFR signaling in NSCLC spheroids shows SOS1 is a therapeutic target in EGFR-Mutated cancer. eLife 9, e58204. 10.7554/eLife.58204 32897190 PMC7478890

[B88] Theard P. L. Linke A. J. Sealover N. E. Daley B. R. Yang J. Cox K. (2024). SOS2 modulates the threshold of EGFR signaling to regulate osimertinib efficacy and resistance in lung adenocarcinoma. Mol. Oncol. 18, 641–661. 10.1002/1878-0261.13564 38073064 PMC10920089

[B89] Tria S. M. Burge M. E. Whitehall V. L. J. (2023). The therapeutic landscape for KRAS-mutated colorectal cancers. Cancers 15, 2375. 10.3390/cancers15082375 37190303 PMC10136970

[B90] Xiao F. Wang K. Wang X. Li H. Hu Z. Ren X. (2024). Discovery of RGT-018: a potent, selective, and orally bioavailable SOS1 inhibitor for KRAS-driven cancers. Mol. Cancer Ther. 23, 1703–1716. 10.1158/1535-7163.MCT-24-0049 39087485

[B91] Yadav K. K. Bar-Sagi D. (2010). Allosteric gating of Son of sevenless activity by the histone domain. Proc. Natl. Acad. Sci. 107, 3436–3440. 10.1073/pnas.0914315107 20133694 PMC2840464

[B92] Yaeger R. Mezzadra R. Sinopoli J. Bian Y. Marasco M. Kaplun E. (2023). Molecular characterization of acquired resistance to KRASG12C–EGFR inhibition in colorectal cancer. Cancer Discov. 13, 41–55. 10.1158/2159-8290.CD-22-0405 36355783 PMC9827113

[B93] Yang S.-S. Van Aelst L. Bar-Sagi D. (1995). Differential interactions of human Sos1 and Sos2 with Grb2. J. Biol. Chem. 270, 18212–18215. 10.1074/jbc.270.31.18212 7629138

[B94] You X. Kong G. Ranheim E. A. Zhou Y. Zhang J. (2016). Loss of Sos1 inhibits oncogenic kras-induced hyperactivation of wild-type ras during leukemogenesis. Blood 128, 1543. 10.1182/blood.v128.22.1543.1543 27658700

[B95] You X. Kong G. Ranheim E. A. Yang D. Zhou Y. Zhang J. (2018). Unique dependence on Sos1 in Kras (G12D) -induced leukemogenesis. Blood 132, 2575–2579. 10.1182/blood-2018-09-874107 30377195 PMC6293870

[B96] Zak K. M. Waterson A. G. Geist L. Braun N. Hauer K. Rumpel K. (2025). Discovery of small molecules that bind to son of sevenless 2 (SOS2). J. Med. Chem. 68, 2680–2693. 10.1021/acs.jmedchem.4c02007 39818983 PMC11831648

[B97] Zhang S. Zhang Y. Chen X. Xu J. Fang H. Li Y. (2022). Design and structural optimization of orally bioavailable SOS1 inhibitors for the treatment of KRAS-Driven carcinoma. J. Med. Chem. 65, 15856–15877. 10.1021/acs.jmedchem.2c01517 36384290

[B98] Zhao W. Jamshidiha M. Lanyon-Hogg T. Recchi C. Cota E. W. Tate E. (2017). Direct targeting of the ras GTPase superfamily through Structure- based design. Curr. Top. Med. Chem. 17, 16–29. 10.2174/1568026616666160719165633 27530972

[B99] Zhou Z. Zhou G. Zhou C. Fan Z. Cui R. Li Y. (2023). Discovery of a potent, cooperative, and selective SOS1 PROTAC ZZ151 with *in vivo* Antitumor efficacy in KRAS-Mutant cancers. J. Med. Chem. 66, 4197–4214. 10.1021/acs.jmedchem.3c00075 36897932

